# Lived Experience, Family, and Clinician Perspectives on Barriers to Adult Autism Diagnosis and Post-Diagnostic Supports: A Mixed-Methods Systematic Review

**DOI:** 10.1007/s10882-026-10055-x

**Published:** 2026-03-05

**Authors:** Valeria Khudiakova, Jacqueline Sin, Mimi Suzuki, Kirsten Barnicot

**Affiliations:** School of Health and Medical Sciences, City, University of London, London, United Kingdom

**Keywords:** Autism, Diagnosis, Post-diagnostic supports, Adulthood, Misdiagnosis, Lived experience

## Abstract

Obtaining a late Autism diagnosis and post-diagnostic supports can be a long, complicated process. Prior research has explored the perspectives of those with lived experience, family members, and clinicians, but has not yet provided a comparison of their perspectives. This review integrates qualitative and quantitative literature on barriers to diagnosis and post-diagnostic supports from these three perspectives. We also examine barriers reported by gender-diverse and ethnic minority Autistic people. We searched Web of Science, PsycINFO, Embase, Medline, and ProQuest Dissertations & Theses. Study quality was evaluated using Joanna Briggs tools and the Delphi Critical Appraisal Tool. We followed mixed-methods integrative methodology and thematic synthesis approaches to synthesise findings, and GRADE-CERQual to assess confidence in the evidence generated. We included 50 studies on 4487 adult-diagnosed participants, 902 self-identifying participants, 217 family members, and 198 clinicians. Ethnicity and gender identity were scarcely reported. Three analytical themes emerged: recognition and decision-making; negotiation at the interface; and resistance and resolutions. Tensions between stakeholders were seen in several areas, including conflicting views on access to information, diagnostic tools, self-identification, and challenges posed by co-occurring mental health conditions. The GRADE-CERQual appraisal showed moderate-to-high confidence in the findings. Barriers were compounded for gender-diverse and ethnic minority participants. Differences in how barriers (e.g., perceptions of diagnostic practices) are interpreted by stakeholders may themselves hinder access to services. We outline research and clinical implications, including transparent demographic reporting and improved access to information about services.

**Supplementary Information:**

The online version contains supplementary material available at https://doi.org/10.1007/s10882-026-10055-x.

## Background

A growing number of adults whose Autism^1^ was not recognised or diagnosed in childhood are now receiving a diagnosis, described as the ‘lost generation’ (Lai & Baron-Cohen, 2015). Many of these adults have extensive histories of involvement with the mental health system, often marred by unsatisfactory and ineffective treatment, while the underlying Autism-related challenges remain unrecognised (Bargiela et al., 2016; Kentrou et al., 2024; Shaw et al., 2022). Although not a cure-all, diagnosis can offer validation, access to support, and a framework for reinterpreting one’s experiences (Bargiela et al., 2016; Shaw et al., 2022). Those with lived experience also report that a late diagnosis can facilitate authenticity and community connectedness, both of which may promote coping and mental well-being post-diagnosis (Botha, 2020; Seers & Hogg, 2023).

The journey to receiving a diagnosis in adulthood and accessing post-diagnostic supports is rarely straightforward. Commonly reported barriers to receiving a diagnosis are difficulties in obtaining referrals, financial and logistical factors, lack of awareness of non-stereotypical presentations, camouflaging (i.e., behaviours aimed at reducing the perception of Autistic traits), and lengthy wait-times (Ardeleanu et al., 2024; Nayyar et al., 2025; Wigham et al., 2023). Yet evidence regarding who is most impacted by these barriers, and how they can be addressed, remains fragmented.

A large proportion of late-diagnosed Autistic adults carry co-occurring mental health diagnoses (Jadav & Bal, 2022). These may be genuine misdiagnoses of their Autistic traits (Au-Yeung et al., 2019), stem from the stress and trauma of living as an Autistic person in neurotypical society (Bradley et al., 2021; Tamilson et al., 2025), or represent separate areas of need (Camm-Crosbie et al., 2019; Shaw et al., 2022). Co-occurring conditions can complicate the identification and diagnosis of Autism due to overlap in traits (Cumin et al., 2022), thus exacerbating disparities in service access for many adults.

The concept of intersectionality offers a crucial lens to study barriers to Autism diagnosis and post-diagnostic support by examining the way that different social identities, including race, ethnicity, gender, social class, mental health challenges, and neurodivergence, can overlap and interact, creating compounding experiences of oppression (Butler et al., 2025; Crenshaw, 1989). In particular, Autistic people who are gender-diverse or from ethnic minority communities are significantly underrepresented in research, which leads to an incomplete understanding of their challenges in accessing supports (Botha and Gillespie-Lynch 2022; Davies et al. 2024b; Malone et al. 2022). Research in other areas of healthcare suggests that ethnic minority and gender-diverse individuals are often deterred from accessing services due to mistrust, concerns about discrimination, and past negative experiences with healthcare providers (Cruz, 2014; Gary, 2005). Understanding how ethnicity or gender-diverse identity may play into the process of seeking an adult Autism diagnosis would help identify potential tailored solutions, while practices such as contextual sensitivity and tailored communication can improve care for all service users (Lauwers et al., 2024).

An adult Autism assessment is, ultimately, a collaborative process, involving those seeking a diagnosis, clinicians, and frequently family members (Wigham et al., 2022). Tensions between Autistic people, families, clinicians, and Autism researchers are well-documented, notably in relation to issues of conceptualisation of Autism and support priorities (Akbulut & Bird, 2025; Bertilsdotter Rosqvist et al., 2023; Pellicano et al., 2014). The resulting polarisation may impede progress on mutually relevant issues and result in the exclusion of dissenting perspectives, including those from marginalised communities (Botha & Gillespie-Lynch, 2022; Dwyer et al., 2025). These dynamics can deepen mistrust and hinder help-seeking (Cumin et al., 2022; Friedman et al., 2024). Research in other areas of medicine suggests that understanding the perspectives of all stakeholders can enhance satisfaction with services, which may be relevant to adult Autism services (Calvard et al., 2023).

## Rationale

This systematic review aims to integrate research on lived experience, family member, and clinician perspectives on barriers to late Autism diagnosis and post-diagnostic supports. There is a substantial body of siloed research that, if synthesised, could help identify areas of agreement, tension, and lack of representation in current research. We additionally review barriers related to ethnicity or gender-diverse identity, as these have not yet been synthesised systematically, despite ethnic minority and gender-diverse adults reporting increased barriers to diagnosis (Ardeleanu et al., 2024; Tien et al., 2025).

At the time of writing, five systematic reviews on related topics had been published, but all were different in scope. One (Lockwood Estrin et al., 2021) focused on girls and women under 21 years old. Another (Howes et al., 2021) explored only clinician perspectives on Autism assessments across ages. Huang et al. (2020) and Nayyar et al. (2025) examined a wider range of experiences of late-diagnosed Autistic people; as a result, barriers to diagnosis and support were not explored in detail. Finally, Kiehl et al. (2024), in addition to having a similarly broad remit, synthesised solely qualitative data on access to diagnosis. None of these reviews integrated the perspectives of multiple stakeholders, examined how ethnicity and gender diversity intersected with the diagnostic process, or included quantitative studies.

Our review integrates both quantitative and qualitative studies to understand factors impeding access to diagnostic and post-diagnostic support for late-diagnosed Autistic people. We use the terms ‘late-diagnosed’ and ‘adult-diagnosed’ interchangeably to refer to people diagnosed with autism for the first time at or after age 16. Additionally, we include the perspectives of self-identifying Autistic adults and those awaiting a diagnosis, as barriers to accessing a diagnosis may explain why they remain undiagnosed. For brevity, we refer to these participants, along with those diagnosed aged 16 years or above, as “individuals with lived experience” or “Autistic people”.

We address the following objectives in this review:

1. Synthesise available literature on the barriers to receiving an Autism diagnosis and accessing post-diagnostic supports in adulthood, as expressed by late-identified Autistic adults, their families, and clinicians.
2. Explore the intersection of ethnicity and gender-diverse identity with experiences of seeking and receiving an Autism diagnosis in adulthood.

## Methods

We adhered to the Preferred Reporting Items for Systematic reviews and Meta-Analyses (PRISMA; Page et al., 2021) and the Enhancing Transparency in Reporting the Synthesis of Qualitative Research (ENTREQ; Tong et al., 2012) statements. Both completed checklists are included in the Supplementary Materials. We also preregistered the review protocol on PROSPERO (CRD42024594639). Ethics approval was not sought as we relied solely on previously published research.

### Search Strategy

We searched Web of Science, PsycINFO, Embase, Medline, and ProQuest Dissertations & Theses, without limit on publication date, on 23 October 2024, repeated on 23 March 2025, using the following combined key terms:

1. autis* or asperger* or ASD or “autism spectrum disorder” or “autistic spectrum disorder” or ASC or “autism spectrum condition” or “autistic spectrum condition” or PDD or “pervasive developmental disorder”.
2. (adult diagnos*) or adult-diagnos* or (late diagnos*) or (late identif*) or (adult identif*).

The tailored search strategies for each database are provided in Appendix A. We additionally performed backwards and forwards citation searching on any relevant reviews and on all included literature.

### Study Selection

The search results were imported into Rayyan (Ouzzani et al., 2016) and de-duplicated automatically. The lead author screened the titles and abstracts against the eligibility criteria, as summarised in Table 1 below. 25% of the identified studies (titles and abstracts) were independently screened by MS (Cohen’s kappa = 0.80). Both reviewers independently assessed the full texts of articles that had passed the screening (Cohen’s kappa = 0.67). Disagreements were resolved by discussion with the wider team.

**Table 1:** Inclusion and Exclusion Criteria

Inclusion	Exclusion
Primary, peer-reviewed quantitative and qualitative research studies	Reviews, editorials, conference abstracts
Reporting on barriers^1^ to accessing an Autism diagnosis or any related post-diagnostic support services.	Not reporting on barriers; support services that do not immediately follow an Autism diagnosis.
Sample includes people seeking or obtaining an Autism diagnosis in late adolescence or adulthood (≥ 16 years old) or self-identifying as Autistic; their family members; clinicians providing Autism assessments or referrals for people after the age of 16. If any other groups are included, data must be reported separately for the groups of interest.	Sample consists entirely of children and adolescents < 16 years old, those diagnosed with Autism before the age of 16, or non-Autistic people; or data on those groups is aggregated with data on people seeking or obtaining a diagnosis after the age of 16.
Available in English	Not in English
	Duplicate data
	Full-text unavailable

^1^Barriers were defined as any factors that could hinder or delay access to an Autism assessment, an Autism diagnosis where appropriate, or post-diagnosis supports. In quantitative studies, variables considered to constitute potential barriers to diagnosis included factors associated with the age of Autism diagnosis in late adolescents and adults, with a late as opposed to early Autism diagnosis, or with wait times for an Autism diagnosis in late adolescents and adults

We used a cutoff of 16 years for a ‘late’ or ‘adult’ diagnosis. This choice recognises that by late adolescence, many will have already formed a stable sense of identity while engaging in self-exploration (Klimstra et al., 2010), thus suggesting similar motivations for seeking a diagnosis between late adolescents and adults.

We extracted data on study characteristics (design, country, sample size), key participant demographics (ethnicity, gender and gender identity, co-occurring diagnoses) and reported barriers to diagnosis and post-diagnostic support. For the second objective, we further examined the barriers reported by ethnic minority or gender-diverse participants among the included studies, as well as any reported barriers specifically related to ethnicity or gender diversity.

### Quality Assessment

The lead reviewer used JBI Checklists specific to included study designs, namely the checklists for Qualitative Research (Lockwood et al., 2015), Case Series (Munn et al., 2020), Case Reports (Moola et al., 2020), and Analytical Cross-Sectional Studies (Moola et al., 2017), as well as the Delphi Critical Appraisal Tool (Grant & Khodyakov, 2025) for the quality assessment. We did not exclude studies based on the quality assessment, as studies with methodological limitations could provide conceptually useful insights (Melia, 2010).

### Data Transformation

We followed a convergent integrated approach, which involves data transformation prior to synthesis (Stern et al., 2020). In addition to presenting qualitative and quantitative data in a joint display (see below), we qualitised quantitative data by transforming it into narrative descriptions according to meaning. For instance, we highlighted the number or proportion of study participants reporting a particular barrier (therefore indicating its relative importance), as well as relationships between relevant variables, such as wait times for a diagnosis and clinical characteristics (Purssell & McCrae, 2024). If a study showed that late-diagnosed participants were statistically more likely than early-diagnosed participants to report a particular characteristic (e.g., certain mental health diagnoses, as was the case in Jadav & Bal, 2022), we qualitised such findings as a potential barrier to diagnosis and included them in the synthesis accordingly.

### Synthesis Methods

Following the transformation of quantitative findings, we used thematic synthesis (TS) across the extracted data (Barnett-Page & Thomas, 2009; Thomas & Harden, 2008). The synthesis was performed on a single data pool consisting of both qualitative and qualitised quantitative data, both of which informed theme development and interpretation concurrently (Heyvaert et al., 2013; Purssell & McCrae, 2024). TS emphasises both interpretation and fidelity to the empirical data, which makes it a recommended method for review questions relevant to clinical practice and decision-making (Booth et al., 2018).

### Coding and Synthesis Steps

Full texts of all included studies were imported into NVivo 14. The lead reviewer, with regular supervision, applied the three stages of TS (Thomas & Harden, 2008):

1. Direct quotes from participants, authors’ interpretations, and qualitised quantitative data (all brought together for the purposes of analysis) were coded according to their meaning using NVivo. Subsequent studies were assigned extant or new codes, as appropriate.
2. The generated codes were inductively collated into descriptive themes by shared thematic meaning. We did not specify any themes prior to analysis but instead identified and interpreted emerging patterns. Where studies reported findings specific to ethnic minority or gender-diverse participants, these were extracted and analysed separately following the main synthesis.
3. We identified conceptual links and patterns amongst the descriptive themes to generate analytical themes. Themes were initially conceptualised by the lead author then refined during discussions with the wider research team until consensus on theme content and interpretation was reached.

We then performed a GRADE-CERQual assessment of the findings (Lewin et al., 2019).

#### Data Integration

Integrating qualitative and quantitative findings allows to go beyond what each component offers by itself, which is particularly relevant when examining the perspectives of multiple stakeholders (Åkerblad et al., 2021; Crump & Logan, 2008). In our synthesis, we combined qualitative and qualitised quantitative data (Stern et al., 2020). We also presented the qualitative and (original) quantitative findings side-by-side in a joint display (Fetters et al., 2013; Fetters & Guetterman, 2021). We extended the structure proposed by Younas et al. (2020) by separating the three perspectives within the qualitative findings section, before interpreting areas of concordance or discordance.

## Results

### Study Characteristics

**Fig. 1:**
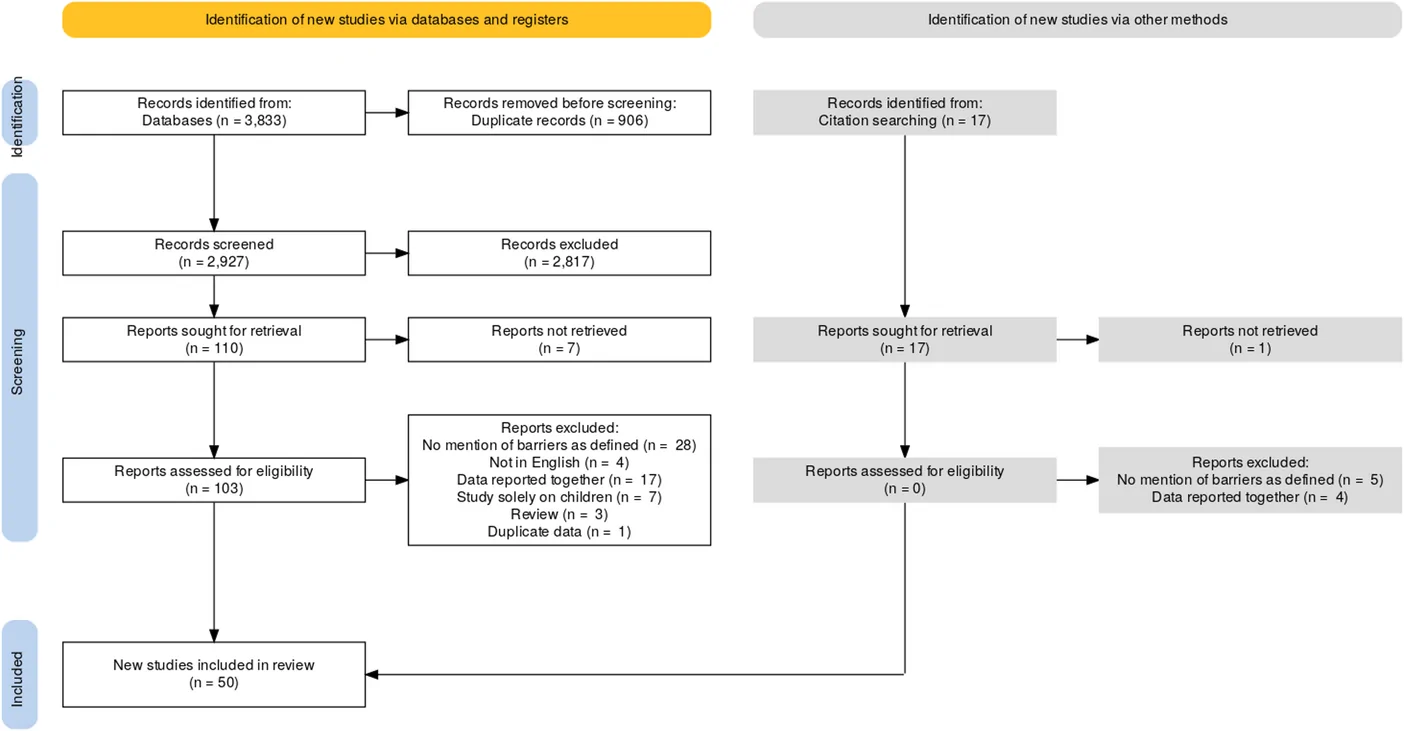
Prisma flowchart

Overall, 50 studies were included. The PRISMA flowchart is provided in Fig. 1, and study characteristics are reported in Appendix B.

Studies contained 9089 participants, including 8382 Autistic participants. Of these, 4487 were diagnosed after the age of 16 years and 902 were self-identifying or seeking an assessment, yielding a total of 5389 lived experience participants included in this review. Additionally, 217 were family members (parents, spouses, or siblings) and 198 were clinicians.

Among the included Autistic participants, 3142 were reported as women or assigned female at birth (AFAB), 1834 were identified as men or assigned male at birth (AMAB), and 330 were gender-diverse. However, some studies did not provide a separate gender breakdown. Eleven studies did not specify if they were reporting on gender or sex assigned at birth.

Thirty-eight studies included data solely from those with lived experience, three solely from family members, and two exclusively from clinicians. In addition, four studies reported on both those with lived experience and their family members. Three included data on all three groups. Thirty-five studies only mentioned barriers to diagnosis, while three were solely focussed on post-diagnostic supports. Twelve reported on both. Twenty-three studies were conducted in the UK, followed by eight in Australia, six in the United States, two each in Canada and the Netherlands, and one study each in Denmark, Brazil, Hong Kong, Italy, Norway, and South Africa. Four studies used multi-national samples.

Thirty studies were qualitative (28 interviews, one conversation analysis, and one open-response survey). Five were case series, two were case studies, and two used Delphi methodologies. Two studies featured both quantitative and qualitative data. Eight studies were quantitative cross-sectional.

### Quality Assessment

Overall, the quality assessment suggested that all studies were of a reasonably good standard. The full ratings, with total scores for each included study, are provided in the Supplement (Tables S.3—S.7). All studies had appropriate designs, analysis methods, and conclusions. However, 54.5% (18/33) of the qualitative studies did not include the authors’ positionality. Only three out of five case series reported how cases were selected. Quantitative studies did not always verify participants’ autism diagnoses, which resulted in lower scores in relation to exposure measurement (although that is less relevant for self-identifying participants).

Among the included qualitative studies, the lowest-scoring studies (all 6/10 on the corresponding JBI checklist) were Cuda Pierce (2022), Pearse (2020), and Punshon et al. (2009). Upon review, these studies did not differ thematically from studies receiving higher ratings, and were largely congruent with the overall thematic synthesis, despite some variation in the depth of reporting due to differences in methodology. A case series by Darling Rasmussen (2023), which contributed to several themes within the synthesis, received a lower score (4/9) primarily due to unclear reporting of the participant selection strategy, though the barriers identified largely aligned with those reported in other studies. Finally, it is noteworthy that studies contributing the most quantitative data to the synthesis (Lewis, 2017; Wigham et al., 2022, 2023) received relatively low scores on the corresponding JBI checklist (4/8). These scores were partly due to issues with exposure measurement (though, as mentioned above, this is of limited relevance given the aims of these studies), unclear outcome measurement in Lewis (2017), and limited reporting of confounds in the remaining three studies. Both Delphi surveys were rated as Moderate quality on the DCAT due to problems with anonymity and controlled feedback. Ultimately, as demonstrated in the GRADE-CERQual assessment, all themes emerging from the synthesis were supported by evidence from higher-quality studies, and no fundamental issues with study design or conclusions were identified across the board.

### Thematic Synthesis of Barriers to Diagnosis and Post-Diagnostic Services

The three analytical themes are presented below, with the descriptive themes provided in the Supplement. All themes were expressed by those with lived experience (LX), family members (FM), and clinicians (CL), though certain findings were more relevant to some groups than others. ‘Interpretation’ refers to interpretations made by primary study authors. Barriers to diagnosis and post-diagnostic supports were explored in all themes.

The qualitative and quantitative findings, and their GRADE-CERQual ratings are presented in Table 2. The full results of the GRADE-CERQual assessment are provided in the Supplement (Table S.8).

**Table 2:** Joint display of qualitative and quantitative findings

Theme and grade CERQual rating	Qualitative findings by perspective			Quantitative findings	Inferences
	Lived experience individuals (LX)	Family members (FM)	Clinicians (CL)		
***Theme 1 Recognition and Decision-Making***					
**1.1 Lack of Appraisal of Needs** High Confidence	Difficult to recognise and articulate their needs and seek supports ^a, b,c, d^ Often hindered by self-stigma and negative attitudes from family.^b, d,e, f,g, h^	Recognised their child’s needs but often did not know where and how to seek help.^i, j^	Not reported	Not reported	Agreement, although not expressed by CL.
**1.2 Realising Past Misdiagnoses and Misattributions** High Confidence	Lengthy but unsatisfying histories of accessing mental health supports caused mistrust.^a, b,c, d,g, k,l, m,n^	Concurrence that past diagnoses did not explain all of the person’s needs.^j^	Misdiagnosis perceived as common, often due to lack of clinician awareness.^o, p,r, s,t, u^	63% of LX participants had previous mental health contact.^v^	Agreement.
**1.3 Evaluating the Costs and Benefits of a Diagnosis** Moderate Confidence	Benefits to receiving a diagnosis were sometimes seen as scarce.^b, k,w, x,y, z,aa^ Perceived risk of discrimination.^b, k,z, aa^	‘Labelling’ sometimes seen as undesirable.^j^	Recognised a diagnosis may not always result in tangible impacts.^o^	63% of LX respondents were concerned about having a diagnosis on their record.^b^	Partial agreement: risks discussed mostly by LX.
**1.4 Community Narratives and Informal Networks** Moderate Confidence	Community knowledge about autism helped recognise their needs and affected decisions about services. ^b, e,z, aa, ad, af, ag^ Autistic spaces were sometimes beneficial but not always safe or desired.^w, ad, ah^	Autistic and/or carer spaces were often beneficial for learning and social connections.^i, j^	Past research was common and often helpful but not always reliable or appropriate.^o^	Not reported.	Partial agreement: benefits of community knowledge acknowledged, but CL raised concerns about reliability.
***Theme 2: Negotiation at the Interface***					
**2.1 Past Negative Experiences and Mistrust** High Confidence	Past negative experiences caused mistrust and a fear of being denied support, especially in those who appeared less ‘stereotypical’.^b, g,k, l,af^	Felt blamed in the past but did not report mistrust.^j, ab^	Recognised the negative effects of past service contact on trust.^o, p^	Mistrust of clinicians was a barrier for 76% of participants.^b^	Partial agreement: FM did not report mistrust, although all groups recognised the effects of past negative experiences.
**2.2 Obtaining a Referral** High Confidence	Referrals difficult to obtain, often refused due to perceived preconceptions.^b, m,af, a.i.^ Information about referrals and services challenging to find.^b, w,ac^	Also struggled to access referrals for their loved ones and find information. ^j, ab, ac^	Recognised the challenges; some advocated for self-referral pathways.^o, p,aj^	27% of LX and 40% of FM had issues obtaining a referral.^v^ 29% of LX and 19% of FM felt that information about services was easy to find compared to 44% of CL.^ac^	Partial agreement: qualitative findings concordant but quantitative findings showed this only applied to a minority. Information about referrals more accessible to CL.
**2.3 The Weight of Expectations** High Confidence	Assessment perceived as high stakes, with fears of a negative outcome.^b, e,k, z,aa^	Not seen as high stakes but a continuation of a lifelong search for explanations.^j^	Expectations amplified by past negative experiences and long waitlists.^o^	Fear of not being diagnosed was reported as a barrier by 87% of participants.^b^	Partial agreement: LX and CL but not FM agree on the emotional weight.
**2.4 Finding the Right Words** High Confidence	Many struggled to articulate their challenges, though those communicating more typically were often dismissed.^b, g,y, af^	Not reported.	Challenges in communication could cause autism to be missed or misdiagnosed. ^p, s,t, u^	88% of respondents named communication challenges as a barrier.^b^	Agreement, although not reported by FM
**2.5 Camouflaging at the Interface** Moderate Confidence	Camouflaging was perceived to cause dismissal, especially in women/AFAB, and pose challenges in post-diagnostic supports.^x, d,e, k,l, aa, af, an^	Not reported.	Some aware of camouflaging and accounted for it during assessments.^o^	Adult-diagnosed participants reported more significant camouflaging in some^ak^ but not all studies.^al, am^	Partial agreement: Seen as a barrier, but unclear if it is specific to late-diagnosed people.
**2.6 Diagnostic Procedures** High Confidence	Mistrust of diagnostic criteria and tools due to perceived bias was common.^f, k,z, aa, af, ah, an^	Many satisfied with the depth of the process.^j^	Current tools perceived as flawed, so discretion played a greater role.^p, aj^	Not reported.	Partial agreement: LX and CL agreed that tools were often flawed but proposed solutions differed. FM discordant.
**Theme 3: Resistance and Resolutions**					
**3.1 Reconciling Complex Explanations** High Confidence	Past diagnoses sometimes complicated recognition of autistic traits as distinct.^e, af^ Informants could provide helpful insights but sometimes were not reliable or accurate.^k, w^	Not reported.	Challenging to disentangle potential autism from co-occurring conditions due to overlap in traits. ^o, p,t, s,u, as, at^ Informants seen as crucial for developmental history but sometimes not feasible.^o, as^	Co-occurring conditions more commonly reported by adult-diagnosed than child-diagnosed participants.^am, ao, ap^ The presence of some co-occurring conditions was associated with shorter wait-times.^ar^	Partial agreement: agree on the utility of informants and challenges in distinguishing autistic traits from co-occurring conditions, although LX more confident in autistic identity.
**3.2 Mental Health Crises** Moderate Confidence	Crises both delayed and facilitated autism recognition and also perceived as secondary to undiagnosed autism. ^d, k,n, z,ag^	Not reported.	Crisis experiences could ‘unmask’ but also mimic traits of autism. Delaying assessment was recommended.^o, as^	Not reported.	Partial agreement: crises could escalate need but disagreed on delaying autism assessment.
**3.3 Systemic Lack of Services** Moderate Confidence	Services for adults were lacking and challenging to locate. Delays common. ^a, b,c, d,k, x,z, aa, ah^	Difficult to find diagnostic and post-diagnostic services. Delays common.^i, j, ab^	Services often seen as inefficient and fragmented.^o, aj^	40% of late-diagnosed autistic people and 36% of family members waited for seven months or longer for their first assessment.^v^ 61% of CL were aware of appropriate local diagnostic services.^ac^	Agreement, though quantitative findings suggested only a minority experienced lengthy waitlists. CL less affected by information barriers.
**3.4 Service Siloing and Fragmentation**	Services were described as fragmented, siloed and confusing.^k, v,ae^	Also struggled to navigate fragmented services.^j, ab, v^	Reported less frequently but some evidence of frustration due to siloing.^p, v^	Not reported quantitatively	Agreement, but more detail reported by LX and FM.
**3.5 Financial and Logistical Barriers** High Confidence	Services often unaffordable, geographically inaccessible, or placed too many demands. ^a, ad, ae, au, av, aw^	Services seen as underfunded and difficult to access.^i, j,ae, au, av^	Services seen as underfunded.^aj, o^	Cost reported as a barrier by 73% of participants.^b^	Agreement, but more detail reported by LX and FM.
**3.6 Invisibility Post-Diagnosis** High Confidence	Needs continued to be dismissed, and there was a lack of appropriate services and supports. ^c, d,h, aa, ad, ae, an, au^	Needs were unrecognised even post-diagnosis.^i, j,ae, au^	Not reported qualitatively	63% of clinicians offered support but only 32% of LX were able to access support post-diagnosis.^v, at^ Only 33% of LX and 30% of FM experienced any recommended post-diagnostic supports, despite them being offered by 61% of clinicians.^ac^	Partial agreement: LX and FM struggled to find and access supports, even though most CL reported offering them.

^a^Ghanouni and Seaker (2023), ^b^Lewis (2017), ^c^Evans (2022), ^d^Leedham et al. (2020), ^e^Atherton et al. (2022), ^f^Friedman et al. (2024), ^g^Punshon et al. (2009), ^h^Lupindo et al. (2023), ^i^Freeman and Paradis (2023), ^j^Raymond-Barker et al. (2018), ^k^Ardeleanu et al. (2024), ^l^Bargiela et al. (2016), ^m^Stagg and Belcher (2019), ^n^Tamilson et al. (2025), ^o^Cumin et al. (2022), ^p^Darling Rasmussen (2023), ^r^Bakken and Høidal (2014), ^s^Fiori Nastro et al. (2023), ^t^Roy & Balaratnasingam (2010), ^u^Smith (2021), ^v^Wigham et al. (2022), ^w^de Broize et al. (2022), ^x^Lam et al. (2024), ^y^Cuda Pierce (2022), ^z^Tien et al. (2025), ^aa^Cage et al. (2024), ^ab^Legg et al. (2023), ^ac^ Scattoni et al. (2021), ^ad^Huang et al. (2024a), ^ae^Finch et al. (2022), ^af^Murphy et al. (2023), ^ag^ Gerhand (2020), ^ah^Seers & Hogg (2023), ^a.i.^Pearse, 2020, ^aj^Hayes et al. (2021), ^ak^McQuaid et al. (2022), ^al^Dodd (2022), ^am^Gonçalves-Garcia et al. (2025), ^an^Craddock (2024), ^ao^Jadav and Bal (2022), ^ap^Dubreucq et al. (2023), ^ar^ McKenzie et al. (2015), ^as^Gilmore & Hayes (1996),^at^Heijnen-Kohl et al. (2025), ^au^Wigham et al. (2023), ^av^ Hamdani et al. (2023). ^aw^ Powell and Acker (2016)

### Analytical Theme 1: Recognition and Decision-Making

This theme details the process of recognising one’s needs and deciding to seek a diagnosis or post-diagnostic supports. Autistic people, and sometimes their families, frequently relied on their own knowledge and understanding of their needs, desired support, and any potential barriers. However, their appraisal of their needs was sometimes hindered by preconceptions and difficulties in recognising and articulating their need for support. At times, there was a mismatch between Autistic people and their clinicians’ perception of their needs. Information about services and how to navigate the healthcare system was often inaccessible, which complicated the process of recognition.

#### Lack of Appraisal of Needs

An awareness of one’s needs as warranting formal support was the first step in deciding to seek an Autism assessment for many Autistic people and their family members. Ghanouni and Seaker interpret this as “noticing a need” (2023). At this stage, some Autistic people struggled to recognise and express their needs and challenges or “[know] what’s normal and what’s not” (LX, Lewis, 2017) even to themselves: “I was self-aware enough to know that there was something wrong” (LX, Evans, 2022). This was often due to an internalised “negative view of mental differences” (LX, Lewis, 2017). Previous harmful messages from others had resulted in “negative self-talk” (LX, Cage et al., 2024), or feeling “wrong, “broken,” or “bad” (LX, Leedham et al., 2020), which had to be overcome to seek a diagnosis. On the other hand, family members had often felt “most of their lives that there was something different about their child” (interpretation, Raymond-Barker et al., 2018).

Autistic people reported that family attitudes hindered their pursuit of an adult diagnosis. These included preconceptions about “the label and [being] put in a box” (LX, Lewis, 2017) or a lack of recognition of their needs: “when I told my dad […] he [said] ‘You don’t have autism, you’re perfect.” (LX, Leedham et al., 2020). Some family members reported a limited awareness of the scope of practice of mental health professionals, such as having “no idea about psychologists” (FM, Freeman & Paradis, 2023).

Challenges in identifying and advocating for one’s needs extended to post-diagnostic supports. Some mentioned a “difficulty knowing ‘who to ask’ or ‘where to go’ for accommodations” (interpretation and LX quotes, Cage et al., 2024). At times, there was misalignment in areas of focus for support between Autistic people and their clinicians: “I found it distressing that the psychologist immediately wanted to start working on my ability to read social cues when it’s the least of my problems at this point” (LX, de Broize et al., 2022).

#### Past Misdiagnoses and Misattributions

A significant proportion of Autistic people (e.g., 63% of late-diagnosed Autistic respondents in Wigham et al., 2022) had previously sought help from the mental health system for their challenges and received other diagnoses.^2^ The process was at times “lifelong” (LX, Punshon et al., 2009).

Autistic people, alongside their family members, were aware that their experiences did not always align with the diagnoses they had received (Raymond-Barker et al., 2018; Tamilson et al., 2025), and how these diagnoses “failed to account for other struggles in [their] daily life” (interpretation of LX, Pearse, 2020). However, it was sometimes difficult to understand why prior diagnoses did not feel right: “I never really found a satisfactory answer” (LX, Punshon et al., 2009). Therefore, there was doubt in their “own judgements even when they knew the suggested diagnoses did not adequately describe or help them” (interpretation of LX, Leedham et al., 2020).

At that time, Autistic people reported that their clinicians had viewed their diagnoses as adequate: “We’re still going to act like that’s the solution, even though you’re communicating that it’s not” (LX, Ardeleanu et al., 2024). Family members concurred with these experiences: “I knew there was something else… and I mentioned it to [clinicians] and nobody ever picked it up because they didn’t have the knowledge themselves” (FM, Raymond-Barker et al., 2018). As a result of misdiagnosis, Autistic people felt “pigeonholed” (LX, Lewis, 2017) and disillusioned with the mental health system: “I got so used to people being so rubbish at diagnosis” (LX, Punshon et al., 2009).

Sometimes, misdiagnoses resulted in “the wrong treatment and medicine” (LX, Ghanouni & Seaker, 2023) or “inappropriate referrals” (interpretation of FM, Raymond-Barker et al., 2018) to services, until Autism was considered. Clinicians recognised the consequences of labelling and unsuitable treatment: “[Autistic women have] obviously been willing to look at issues and explore ways of improving their lives, but they just can’t seem to get out of it” (CL, Cumin et al., 2022).

Autistic people and clinicians lamented experiences of “lazy” misdiagnosis (LX, Tamilson et al., 2025), especially with highly stigmatised conditions such as borderline personality disorder (BPD), which was, notably, exclusively discussed in relation to women/AFAB individuals. A BPD (mis)diagnosis was often based on limited evidence yet overshadowed other concerns: “Much weight had initially been put on her descriptions of uncontrollable anger and self-harming, but not much attention had been given to the circumstances” (CL, Darling Rasmussen, 2023). Consequently, the fear of stigmatisation prevented some from seeking an Autism assessment: ‘Because of the BPD diagnosis I was too afraid to go through the [general practitioner [GP]” (LX, Tamilson et al., 2025).

Studies from clinicians’ perspectives concurred with the potential negative impacts of misdiagnosis. Several case studies and case series detailed the journeys of clients that had had extensive involvement with mental health services before autism was suspected or diagnosed (Darling Rasmussen, 2023; Fiori Nastro et al., 2023; Roy & Balaratnasingam, 2010). This was attributed to “a lack of professional awareness of the condition” (CL, Smith, 2021) or direct experience: “training alone is unlikely to result in confidence as diagnostic capability requires exposure to enough cases” (CL, Smith, 2021).

#### Evaluating the Costs and Benefits of a Diagnosis

Ultimately, seeking a diagnosis often depended on Autistic people and their family members’ appraisal of its costs and benefits, with some reporting “no tangible benefits” (LX, Ardeleanu et al., 2024) to receiving a diagnosis in the absence of “assistance or help that [they] would get from it” (LX, Lewis, 2017). The sensory, emotional, and energy burdens of an assessment were also considered: “I had to take hours of public transport, which is very difficult for me from a sensory and anxiety point of view” (LX, de Broize et al., 2022). Some reported that their clinicians also did not see the value in an adult diagnosis: “A psychiatrist… suspected [Autism], but said doing an assessment at such an old age is useless” (LX, Lam et al., 2024), and some clinicians recognised that a diagnosis might not result in practical benefits (Cumin et al., 2022).

Ultimately, some decided that “the risks of a diagnosis outweighed the potential benefits” (interpretation of LX, Ardeleanu et al., 2024) and remained self-identifying. Some were “unsure if [they] will lose any rights with a diagnosis” (LX, de Broize et al., 2022) or were concerned “they wouldn’t be welcome in countries for being autistic… or run into barriers for being able to adopt children” (interpretation of LX, Tien et al., 2025). This was supported by a quantitative finding in Lewis (2017), where 63% of autistic respondents cited concerns about an Autism diagnosis appearing on their record as a barrier to pursuing a diagnosis.

Some recognised that a clinical diagnosis may allow them to have legal protections: “someone says to you, why do you need this reasonable adjustment at work… I have my certificate here” (LX, Cage et al., 2024). Equally, the potential for employment discrimination was a deterrent: “when you have that paper you are forced to tell your boss or you’re committing fraud” (LX, Lewis, 2017). Family members also worried about the stigma associated with the diagnosis: “I was led to believe that it was in Adam’s best interests not to label him…it was thought it would rock the boat” (FM, Raymond-Barker et al., 2018).

#### Community Narratives and Informal Networks

In contrast to information about diagnostic services, Autistic people and clinicians agreed that it was quite easy to find information online about Autism from community-based sources (Cumin et al., 2022; Murphy et al., 2023; Tien et al., 2025). This facilitated “patient research” (interpretation of CL, Cumin et al., 2022) prior to the assessment, which was helpful for some in articulating their needs (LX, Lewis, 2017) but was not always seen as reliable by clinicians (Cumin et al., 2022).

Autistic community narratives sometimes affected decisions about seeking a diagnosis: “I started to see autistic people… talking about their experience” (LX, Tien et al., 2025), or the choice of services. The perceived excessive wait times and low likelihood of obtaining a diagnosis, as per these narratives, led some to avoid public healthcare: “I didn’t want to waste time or hassle with the NHS [UK National Health Service]… They’ll say… ‘Why waste time on you.’” (LX, Atherton et al., 2022). On the other hand, private providers “would not have offered the same level of credibility” (interpretation of LX, Gerhand, 2020).

When accessing post-diagnostic services, both Autistic people and family members valued communities of people with similar lived experiences: “It’s a better environment for her than going to mental health groups… She loves the Aspergers group” (FM, Raymond-Barker et al., 2018). However, some Autistic people experienced stigmatisation and hostility in Autistic-led spaces: “I got bullied out of an autistic mums’ group” (LX, Seers & Hogg, 2023). One Autistic adult with intellectual disability (ID) felt devalued in online Autistic communities: “Just because we may not understand or we take longer to learn or we can’t learn something doesn’t make our value less” (LX, Huang et al. 2024a). Others expressed discomfort at the thought of sharing their experiences with others, as they “wouldn’t want to sit around a group of people and talk about it” (LX, de Broize et al., 2022).

### Analytical Theme 2: Negotiation at the Interface

The ‘interface’ between autistic people, their family members, and clinicians was fraught with stereotypes and attitudes that hindered access to a diagnosis. Autistic people and family members reported that the referring, treating, and assessing clinicians had varying levels of knowledge about Autism (especially in women^3^), which delayed support for many. Some clinicians, however, were aware of these challenges and worked to address them.

#### Past Negative Experiences and Mistrust

Seeking an assessment was commonly a source of “anxiety” (LX, Lewis, 2017). Autistic people anticipated being “misunderstood” (LX, Lewis, 2017) or “invalidated” (LX, Murphy et al., 2023) due to a history of negative interactions with clinicians “who had just failed completely to get to the bottom of what was wrong with me” (LX, Punshon et al., 2009). These fears were amplified in those who felt they contradicted common Autism stereotypes: “I am not obviously autistic so I fear being told by others that I am making it up” (LX, Lewis, 2017).

Lewis (2017) found that 76% of those with lived experience cited mistrust of clinicians as a barrier in seeking a diagnosis. Family members were also “met with reluctance, blaming and dismissive responses from professionals” (interpretation, Legg et al., 2023). Past negative interactions also caused a fear of being denied post-diagnostic supports: “I am always dead paranoid that someone is going to say, ‘Oh we have made a mistake and you haven’t got Asperger syndrome… So you can’t have access to any of the services’” (LX, Punshon et al., 2009).

#### Obtaining a Referral

Requesting a referral for an Autism assessment was described as “frustrating” (FM, Raymond-Barker et al., 2018) or “like batting my head against a brick wall” (LX, Evans, 2022), expressed across genders. These experiences of not being believed were attributed to lack of knowledge of Autism beyond stereotypes: “The GP dismissed me with, “I think if you finished university, you can’t have Asperger’s”” (LX, Seers & Hogg, 2023). One participant in Pearse (2020) had a referral “refused on the grounds that she does not require “support” to cope with daily life” (interpretation + LX). However, quantitative findings in Wigham et al. (2022) suggested these experiences were a minority, albeit a significant one: 27% of their Autistic respondents and 40% of family members struggled to obtain a referral.

Clinicians recognised the difficulties many clients faced in obtaining referrals, with particular reference to women (Hayes et al., 2018; Wigham et al., 2022). Some advocated for the possibility to self-refer to diagnostic services “to bypass gatekeeping by professionals who lack awareness of the varied and subtle ways in which the condition can present’” (CL, Wigham et al., 2022).

During the referral process, information about pathways and resources appeared to be more accessible to clinicians than those seeking a diagnosis. In Scattoni et al. (2021), fewer Autistic people and family members than clinicians felt that information about obtaining an adult diagnosis and locating services was “easy to find”. This lack of guidance extended to navigating “the complexity of the health care system” (LX, Lewis, 2017): “It’s hard to… navigate your way through this whole lot, it’s a labyrinth” (LX, de Broize et al., 2022).

#### The Weight of Expectations

Both Autistic people and clinicians acknowledged the emotional weight and expectations surrounding the assessment. The pressure and the high stakes of the outcome were described as “psychologically draining” (LX, Atherton et al., 2022). Some clinicians believed these expectations were amplified by the “lengthy waitlists” (interpretation, Cumin et al., 2022). However, family members did not feel the same emotional weight, as the Autism assessment “was regarded as a continuation of normative circumstances, as simply another assessment” (interpretation, Raymond-Barker et al., 2018).

Concerns about not receiving a diagnosis were a deterrent to many (87% in Lewis, 2017), who feared “how ‘invalidating’ it would be to be told by a clinician ‘after all this time’ that they did not meet the criteria and how it would be hard to ‘trust their assessment’” especially given previous negative experiences of misdiagnosis in Theme 1.2 (interpretation + LX, Cage et al., 2024). Clinicians also recognised the “far-reaching damage to [the client’s] mental health and trust in the medical system” (CL, Cumin et al., 2022) that could happen if an Autism diagnosis was not made.

#### Finding the Right Words

In Lewis (2017), 88% of participants reported the “inability to adequately communicate symptoms to a provider” as a significant barrier to Autism diagnosis. Autistic participants in Murphy et al. (2023) believed “literal interpretation, black-and-white thinking and difficulty communicating experiences verbally” (interpretation) caused misinterpretation. Some struggled to find words that would adequately convey their experiences: “I only had the words [non-autistic people] use to describe themselves…” (LX, Lewis, 2017). Thus, their clinicians likely saw little reason to consider autism, while the burden to explain their experiences was placed on Autistic people: “[The psychiatrist] said ‘How can I help you if you don’t tell me what is wrong?’” (LX, Punshon et al., 2009). Yet people with seemingly more typical communication behaviours also experienced invalidation due to their clinicians’ assumptions about Autistic communication: “He said, “Oh, no. You’re not autistic… If you had autism, you’d be like looking at your feet. You would not want to look at my face.” (LX, Cuda Pierce, 2022).

Clinicians acknowledged the possibility of misinterpretation. Fiori Nastro et al. (2023) described a patient nearly diagnosed with a psychotic disorder because he had communicated his challenges in “in a paranoid manner,” before being assessed for Autism. Sometimes, atypical communication behaviour was noticed but misattributed—for instance, to offending behaviour (CL, Smith, 2021).

#### Camouflaging at the Interface

Camouflaging of autistic traits sometimes amplified miscommunication between Autistic people and their clinicians. Clinicians might have seen them as “not problematic enough” (interpretation, Lam et al., 2024), thus contributing to their struggle to be believed or recognised. However, quantitative studies exploring whether camouflaging was associated with a late diagnosis were inconclusive: Adult-diagnosed participants in McQuaid et al. (2022) but not Dodd (2022) or Gonçalves-Garcia et al. (2025) reported higher levels of camouflaging than child-diagnosed participants.

“‘Masking’ during the diagnostic process” (interpretation, Cage et al., 2024) was understood to disproportionally disadvantage women: “Boys do it to an extent, but not nearly as much as women” (LX, Murphy et al., 2023). However, some women and AFAB participants reported feeling sufficiently “safe” to “unmask” (LX, Murphy et al., 2023) during the assessment, which ultimately helped them to be recognised. Some clinicians were aware of the possibility of camouflaging complicating autism assessments and took it into consideration (Cumin et al., 2022).

Camouflaging posed similar challenges when accessing post-diagnostic supports: “I feel burdened by having to make those around me aware that I’m not coping, which can be challenging when your outward presentation and behaviour do not reflect your internal experiences” (LX, Craddock, 2024). Interestingly, camouflaging as such was not explicitly discussed by, or in reference to, men/AMAB individuals.

#### Diagnostic Procedures

Many Autistic people distrusted the diagnostic criteria, viewing them as geared towards “a very specific person,” usually understood as a cisgender man (LX, Ardeleanu et al., 2024). Some advocated for an overhaul of diagnostic criteria and “a ‘separate’ list for females” (LX, Craddock, 2024). However, other Autistic women believed there should be a choice of criteria “based on how you felt it would be appropriate” (LX, Murphy et al., 2023). For clinicians, the challenge seemed to be understanding that the criteria subsume “much greater heterogeneity than is realised by many health professionals” (CL, Darling Rasmussen, 2023).

Autistic people and clinicians questioned the validity of diagnostic tools, described as “a bit weird… absurd” (LX, Cage et al., 2024). Women again felt they were not the “target audience” for these tools (LX, Seers & Hogg, 2023). Clinicians described standard tools as lacking “sensitivity” (CL, Hayes et al., 2021) and “unequipped to detect autism in adult women of typical intelligence” (CL, Cumin et al., 2022). For these reasons, some of those who were denied a diagnosis remained steadfast in their Autistic identity: “Although they might not have the official diagnosis, they ‘knew their truth’” (interpretation + LX, Cage et al., 2024). At times, clinicians relied on the “feeling in the room” (CL, Cumin et al., 2022) to counter the limitations of diagnostic tools, especially when they suggested clients were “not at the ‘diagnosis point’” (CL, Hayes et al., 2018). There was little data on family members’ views on the diagnostic tools, yet they appeared “very satisfied” (interpretation of FM, Raymond-Barker et al., 2018) with the process. Some self-identified people perceived the diagnostic process as an undue pathologisation of Autistic identity and refused to engage with it: “Diagnoses related to identity shouldn’t exist” (LX, Friedman et al., 2024).

### Analytical Theme 3: Resistance and Resolutions

While Analytical Theme 2 explores challenges at the interface between the three groups, Analytical Theme 3 examines broader patterns of contradictions and resistance during the path to diagnosis. The diagnostic process sometimes involved reconciling competing or contradictory explanations, especially in relation to co-occurring mental health conditions. Systemic issues in service design and implementation compounded the challenges faced by Autistic people and their families in seeking a diagnosis or post-diagnostic supports.

#### Reconciling Complex Explanations

Compared to child-diagnosed participants, adult-diagnosed participants in Jadav and Bal (2022) and Gonçalves-Garcia (2025) were significantly more likely to report co-occurring conditions. Yet the presence of some co-occurring diagnoses—namely neurological or speech conditions and ID—predicted shorter wait times between referral and assessment in (McKenzie et al., 2015). The resultant wait for a diagnosis was longer due to a greater number of appointments, likely due to said complexity.

Indeed, co-occurring diagnoses caused additional challenges in determining the most appropriate explanation and avoiding misdiagnosis, especially in people with complex presentations. Clinicians described instances of conflation of Autistic traits with other conditions, such as childhood trauma (Cumin et al., 2022), visual impairment (Gilmore & Hayes, 1996), dementia (Heijnen-Kohl et al., 2025), psychosis (Fiori Nastro et al., 2023), ID, or offending behaviour (Smith, 2021). Autistic people also reported challenges in separating potential Autistic traits from those associated with co-occurring conditions: “Maybe I don’t actually have autism… I do just have social anxiety” (LX, Murphy et al., 2023). Symptoms of co-occurring conditions were at times more conspicuous and thus recognised more readily than Autistic traits, while the underlying Autism-related challenges remained unaddressed: “The development of comorbidity may cause further delay in correctly diagnosing and treatment as these symptoms are often more recognisable to the inexperienced eye” (interpretation of CL, Darling Rasmussen, 2023).

To navigate these complexities, many clinicians relied on clients’ developmental history: “without a clear developmental history… it would be easy to diagnose subtypes of schizophrenia instead of autism” (CL, Roy & Balaratnasingam,^2010^). However, there was no consensus on the feasibility and utility of an informant for that purpose. Some felt that informant-provided developmental history “was more honest” (LX, de Broize et al., 2022), as “the individual’s own recall… can be prone to errors” (CL, Smith, 2021), though informants were not always accurate: “It really wasn’t a true observation” (LX, de Broize et al., 2022). It was also acknowledged by clinicians that finding “an external informant who knew them in childhood” (interpretation of CL; Cumin et al., 2022) disadvantaged many, especially older adults^4^ (Heijnen-Kohl et al., 2025; van Niekerk et al., 2011).

#### Mental Health Crises

Urgent mental health concerns, such as a suicidal crisis, often took priority and caused delays in diagnosing Autism: “Figuring out my quirks lost its importance to the professionals once I got suicidal” (LX, Tien et al., 2025). Yet for some, a crisis actually facilitated receiving an Autism diagnosis: “In the end it was like actually, I can’t, I’m done, I’ve had enough” (LX, Gerhand, 2020), as “some patients are not recognised until they have developed severe comorbidity” (CL, Darling Rasmussen, 2023). Some clinicians proposed that because of the trait overlap between Autism and some mental health conditions, the exploration of Autism could be worth postponing to avoid misidentification: “Sometimes the priority is to treat the depression, and then when that’s lifted, to see what’s underneath” (CL, Cumin et al., 2022). Autistic people, across genders/sexes, seemed to disagree with that approach. They perceived their mental health conditions as stemming from “just [living as] an undiagnosed autistic person who had trauma” (LX, Tamilson et al., 2025), which might be partially resolved with an Autism diagnosis and the conferred self-understanding (LX, Leedham et al., 2020; Punshon et al., 2009).

#### Systemic Lack of Services

Generally, adult diagnostic services were scarce: “Everything seemed geared towards children” (LX, Lewis, 2017). When services were available, delays were very common, spanning “years” (LX, Ardeleanu et al., 2024). In Wigham et al. (2022), 40% of late-diagnosed Autistic people and 36% of family members reported having to wait for seven months or longer for the first assessment. The period following the referral was plagued by delays and unnecessary bureaucracy: “The assessment clinic keeps just saying we need more evidence before we can assess you” (LX, Cage et al., 2024).

Locating qualified providers also proved challenging, which was mentioned by Autistic women/AFAB participants in particular: “very difficult, even in [a capital city], to find an adult specialist clinician for autism spectrum disorders” (LX, de Broize et al., 2022). Specialists sometimes lacked adequate knowledge of services in the local area; for instance, in Scattoni et al., 2021, only 61% of clinicians reported having “knowledge of good local service models for autism diagnosis in adulthood.”

These systemic issues did not resolve following diagnosis, as post-diagnostic supports were similarly scarce, causing frustration to Autistic people and their families after having endured a “battle” in obtaining the diagnosis (LX, Evans, 2022). Oftentimes, “there were no services in place for my situation” (LX, Lewis, 2017), with a recognition that “they can’t offer us what’s not there” (FM, Legg et al., 2023).

#### Service Siloing and Fragmentation

In relation to seeking a diagnosis, the lack of integration and communication between different services caused great frustration (LX, FM, and CL, Wigham et al., 2022). As a result, the system was seen as fragmented, with “hoops that most people would not be able to really navigate” (LX, Ardeleanu et al., 2024). Some adults experienced delays from being “referred around from team to team” in pursuit of an assessment (FM, Raymond-Barker et al., 2018).

Family members reported feeling “lost”, “blind” and “in the dark” (FM, Legg et al., 2023) after the diagnosis. These experiences were linked to a lack of (LX and FM, Huang et al. 2024a), “conflicting” (FM, Legg et al., 2023), or “out of date” (LX, Finch et al., 2022) information about services. This was perceived as placing Autistic people at a particular disadvantage: “If I, as a relatively articulate, intelligent woman, could not find help, how on earth can we expect people on the autistic spectrum to find this help?” (FM, Raymond-Barker et al., 2018). Many post-diagnostic services were also inappropriately siloed: “He doesn’t come under our remit because he hasn’t got… educational needs” (FM, Raymond-Barker et al., 2018), which sometimes led to losing previous supports: “The mental health service said they could no longer help me’ (LX, Wigham et al., 2023).

#### Financial and Logistical Barriers

Adult diagnostic services were perceived as underfunded, which resulted in missed opportunities and delays (LX and FM; Finch et al., 2022; FM, Raymond-Barker et al., 2018). For many lived experience and family member participants, the cost of a private diagnosis was “unaffordable” (interpretation of LX and FM, Hamdani et al., 2023), especially for individuals who, “like many autistic people, [were] under employed and not well paid” (LX, Ghanouni & Seaker, 2023). Indeed, 73% of Autistic participants in Lewis (2017) cited financial factors as a barrier to diagnosis. The communication demands of trying to access services additionally disadvantaged those with co-occurring conditions: “Between autism, depression and chronic fatigue…ringing people up in the first place is challenging” (LX, Huang et al. 2024a).

Similar financial and logistical barriers hindered access to post-diagnostic supports: “I live about an hour out of the city and again, they’re very expensive. So unless I’m really, really desperate for mental health services, I’m not going there” (LX, Huang et al. 2024a). This lack of accessibility meant that help was only sought in times of crisis, while Autistic people’s desire to work with a professional “to try to figure how we fit in the world” (LX, Ghanouni & Seaker, 2023) was unmet.

#### Invisibility Post-Diagnosis

Even when post-diagnostic services were available, Leedham et al. (2020) interpreted newly diagnosed Autistic people’s journeys as an “ongoing battle for needs to be recognised by certain services.” The needs of Autistic people were perceived as “invisible” (interpretation of LX and FM, Finch et al., 2022) to service providers: “I think people assume I don’t need help or support” (LX, Huang et al. 2024a). Some were not believed about their diagnosis or needs: “A diagnosis is not an automatic ticket to recognition and acceptance for women” (interpretation of LX, Craddock, 2024), and newly diagnosed men/AMAB individuals reported such challenges as well (LX, Lupindo et al., 2023).

Wigham et al. (2023) and Scattoni et al. (2021) reported that most of their clinician participants routinely offered follow-up appointments or recommended post-diagnostic supports. However, in both studies, only a third of their Autistic respondents had accessed post-diagnostic supports, potentially revealing gaps in provision. Alternatively, the post-diagnostic supports offered by clinicians might not have seemed adequate or accessible to Autistic people.

Social service agencies did not always view Autism as “a significant disability” (LX, Finch et al., 2022), especially in those without an ID: “Adam could get no help from social services because he wasn’t diagnosed with an additional [ID]” (FM, Raymond-Barker et al., 2018). Family members had to fight for supports for their adult children: “Look I am absolutely begging for help now. I am not going to go until I get a referral for help” (FM, Raymond-Barker et al., 2018).

### Barriers Related to Gender Diversity and Ethnicity

As noted earlier, reporting on ethnicity and gender identity was scarce across studies; reported barriers experienced by gender-diverse and ethnic minority participants are summarised in Table 3.

**Table 3:** Barriers Related to Gender Diversity and Ethnicity

	Studies with Directly Traceable Data	Shared Barriers	Unique Barriers	Inferences
Gender Diversity	Ardeleanu et al., 2024; Friedman et al., 2024; Tien et al., 2025.	Fears of dismissal (2.1, 2.2); invisibility of needs and traits (1.1); logistical and financial barriers (3.5); lack of clinician and family awareness (1.1); past misdiagnosis (1.2).	Fears of losing autonomy and experiencing transphobia; mistrust of clinicians’ competence in relation to gender diversity.	Diagnosis often understood as simultaneously beneficial and constraining. Systemic inequalities remain underexplored. Note that reporting of gender/sex was often unclear across studies, and gender-diverse people were only reported on in 12 studies.
**Ethnicity**	Ardeleanu et al., 2024; Tien et al., 2025; Roy & Balaratnasingam, 2010	Low confidence in receiving a diagnosis (2.6); invisibility of needs and traits (1.1); logistical and financial barriers (3.5); lack of clinician and family awareness (1.1); lack of post-diagnostic services (3.3).	Perception that ethnic minority adults are not the target demographic for an autism diagnosis; cultural factors creating scope for misinterpretation.	System perceived as racist but lived experience data is scarce. Lack of awareness of how autistic traits intersect with and are understood in different cultures can contribute to underdiagnosis. Systemic inequalities remain underexplored. Note that ethnicity was reported in only 10 studies.

#### Gender Diversity

Gender-diverse participants were reported in 12 studies, but only three studies reported data that could be directly attributed to this group. Across these studies, gender-diverse participants reported many of the barriers explored previously, including fears of dismissal (Ardeleanu et al., 2024; Friedman et al., 2024); frustrations with systemic factors, clinicians’ misconceptions about Autism, misdiagnosis (Ardeleanu et al., 2024; Tien et al., 2025); financial barriers, family attitudes, delays (Ardeleanu et al., 2024); past camouflaging, not seeing the value in a diagnosis (Tien et al., 2025); self-doubt about the nature of their needs, and disagreeing with the idea of a clinical diagnosis (Friedman et al., 2024).

Concerns about referring and diagnosing providers’ “knowledge and skills” (LX, Ardeleanu et al., 2024) were acutely felt by gender-diverse participants: “a lot of GPs aren’t trained on trans issues so they’ll often just reject trans people because they don’t know about them” (LX, Friedman et al., 2024). Influence of stigmatising public narratives was also named (Friedman et al., 2024). Some struggled to find a trustworthy diagnostician: “I would want to make sure that the person was competent when it comes to gender stuff… I just don’t have faith that that’s what the majority of providers are doing” (LX, Ardeleanu et al., 2024).

Some gender-diverse Autistic people felt that a diagnosis would result in a loss of autonomy, especially in relation to gender-affirming care: “It just opens up more avenues or reasons for a doctor to deny you [gender-affirming] services” (LX, Ardeleanu et al., 2024). One of participants in Friedman et al. (2024) summarised: “There’s a lot of things a diagnosis gives you, but there’s also a lot of things that it takes away.”

#### Ethnicity

Ten studies reported on ethnic minority participants, of which three provided data that can be attributed to ethnic minorities. Ardeleanu et al. (2024) and Tien et al. (2025) included each participant’s ethnicity, allowing to identify quotes from ethnic minority participants, while the entire sample in the case series by Roy and Balaratnasingam (2010) consisted of Australian Indigenous participants. Two additional studies provided data on ethnicity-related barriers to an adult Autism diagnosis. However, this could not specifically be attributed to ethnic minority participants, as ethnicity data was either not reported (Tamilson et al., 2025) or all participants were White (Craddock, 2024).

Where available, ethnic minority participants reported many barriers discussed previously. They named a lack of financial resources, family attitudes, clinician misconceptions, and delays (Ardeleanu et al., 2024) and a lack of accessible post-diagnostic services (Tien et al., 2025). Ardeleanu et al. (2024) described that their participants felt “they did not match the typical demographics of people who oftentimes receive autism diagnoses, namely, young White males.” They thus had no confidence in receiving a diagnosis: “even though I qualified for [autism]… I don’t think I’m getting diagnosed.”

One participant of unreported ethnicity in Tamilson et al. (2025) lamented that ‘the whole [Autism assessment] is racist, classist, sexist.” A White participant in (Craddock, 2024) felt that “sexism/racism [was] utterly engrained in all of our social institutions,” including Autism services.

Roy and Balaratnasingam (2010) acknowledged the role of cultural factors in access to Autism diagnosis. They conjectured that “Indigenous people would be at significant disadvantage as their cultural norms of not making eye contact may mimic [schizophrenia and autism]”, which could plausibly result in under-diagnosis and thus requires a holistic exploration of a client’s context. However, they made little note of the systemic inequities experienced by Indigenous populations. Therefore, it appears that a limited understanding and recognition of Autism in people from diverse cultural backgrounds remains one of the drivers of diagnostic disparities, but research on systemic issues is limited.

## Discussion

Accessing an Autism diagnosis and post-diagnostic supports in adulthood is often challenging. This mixed-methods systematic review synthesised literature on barriers to late Autism diagnosis and post-diagnostic support, as reported by those with lived experience, their family members, and clinicians working with them. We additionally examined the specific barriers faced by Autistic people from ethnic minority or gender-diverse communities.

Overall, our synthesis revealed that all three stakeholder groups agreed that barriers to diagnosis and post-diagnostic services were pervasive and multilayered, falling into three themes: recognition of need and decision-making; navigating hostile interfaces; and resolving resistance and perceived complexity. Yet each group described and experienced them differently.

Access to information was a core area of disagreement. Autistic adults and their family members often struggled to find information about available services (de Broize et al., 2022; Lewis, 2017), which was less commonly reported by clinicians (Scattoni et al., 2021). While these challenges may place many Autistic people at a disadvantage due to executive functioning difficulties (Demetriou et al., 2018) family members also struggled to navigate these systems (Raymond-Barker et al., 2018; Wigham et al., 2022). This points at a systemic issue whereby access to services hinges on ‘insider’ knowledge. Yet concerningly, many clinicians also lacked knowledge of local adult diagnostic services (Scattoni et al., 2021). As many clinicians rely on informal knowledge reinforced via clinician networks in their decision-making (Gabbay & le May, 2016), gaps in access to information across all stakeholder groups can further contribute to the fragmentation of services and deepen inequities in access to diagnosis.

A similar pattern of disagreement was observed in relation to obtaining referrals. Those with lived experience and family members reported difficulties in gaining referrals (Lewis, 2017; Raymond-Barker et al., 2018). However, this issue was less prominently described by clinicians, and quantitative data showed that this only affected a minority of service users and family members (Wigham et al., 2022). It is possible that this discrepancy reflects self-selection effects, whereby qualitative studies may draw those who have experienced more obstacles to diagnosis and are thus more willing to share their struggles, and assessing clinicians would only see service users who had overcome these barriers. Perspectives of GPs, who were often described as gatekeepers (Craddock, 2024; Tamilson et al., 2025), were not represented in this review, so the specific mechanisms hindering referral are not yet documented.

Autistic adults and family members cited mental health difficulties as a catalyst for seeking an Autism assessment, believing that a diagnosis could help provide answers (Leedham et al., 2020; Finch et al., 2022; Raymond-Barker et al., 2018; Legg et al., 2023). However, clinicians emphasised the particular challenge of disentangling autistic traits from symptoms of co-occurring conditions, with the latter often being prioritised for support (Cumin et al., 2022; Darling Rasmussen, 2023). Whether mental health challenges were viewed as consequences of undiagnosed Autism or separate conditions represented another disagreement between lived experience and clinical practice. Therefore, current siloed approaches may not be sufficient to meet the mental health needs of late-diagnosed Autistic people (Jadav & Bal, 2022) thus perpetuating the cycle of diagnostic delays and ineffective supports.

The suggested ways of resolving the described barriers also differed between the stakeholder groups. Those with lived experience and clinicians agreed on the limitations of diagnostic tools and the intense emotions surrounding the assessment (Ardeleanu et al., 2024; Cumin et al., 2022). Autistic people emphasised the viability of self-identification in light of barriers to diagnosis (Cage et al., 2024; Lewis, 2017), yet clinicians tended to be more cautious about overdiagnosis, especially when co-occurring conditions posed complexity (Cumin et al., 2022). Thus, the disagreements reported by those with lived experience and clinicians extended beyond specific tools and practices but to what it *means* to be Autistic and who has the right to make that judgement (Cage et al., 2024; Friedman et al., 2024; Pearse, 2020). Self-identification remains a contentious issue in the literature (Fellowes, 2024), though self-identifying and clinically diagnosed Autistic people tend to report similar patterns of challenges (Sturm et al., 2024). Our findings cannot offer a judgement on the validity of self-identification yet highlight an area of unresolved tension in the literature: understanding what diagnostic ‘accuracy’ means for contested categories like Autism where lived experience may not always align with clinical practice (Friedman et al., 2024; Kourti, 2021).

These tensions suggest that fragmentation of perspectives might itself hinder access to services, and as clinicians hold the power in clinical settings, barriers perceived by Autistic adults will most likely be perpetuated. Crucially, mistrust was a key barrier reported across quantitative and qualitative studies, stemmed from past experiences of dismissal and inappropriate support as well as some community narratives (Ardeleanu et al., 2024; Craddock, 2024; Friedman et al., 2024; Lewis, 2017). Therefore, what is viewed as meaningful and satisfying support (including for mental health conditions) appears to differ substantially, likely giving rise to experiences of dismissal. This misalignment was further highlighted by the discrepancy between the proportion of clinicians offering post-diagnostic services and that of Autistic people accessing them (Scattoni et al., 2021; Wigham et al., 2022) and supported by narratives describing those supports as scarce (Legg et al., 2023). Perhaps, what clinicians offered was not satisfying or accessible to service users and families.

These findings can be understood through the lens of the double empathy theory, which posits that communication gaps between Autistic and non-Autistic people arise from a bidirectional mismatch in communication styles (Milton, 2012), supported by research on the relative ease of Autistic-Autistic interactions (Crompton et al., 2020). During assessments, this mismatch may contribute to a ‘black box’ process, whereby clinician’s rationale is obscure to the client (Edwards & Elwyn, 2006), perhaps further undermining client-clinician communication and trust (Cumin et al., 2022). This is especially concerning, given that Autism assessments tend to show a lack of consistency in the diagnostic process and a significant reliance on clinical judgement, which may lead to less reliable diagnostic decisions (Hayes et al., 2021). Indeed, in our review, many clinicians felt that extant diagnostic tools lacked sensitivity, especially in women and older adults, and thus relied on their intuition (Cumin et al., 2022; Hayes et al., 2018; Heijnen-Kohl et al., 2025), which highlights the need to further probe the extent to which diagnostic tools and practices, and the way their use is communicated, serve the adult population and who may be left disadvantaged.

Our examination of the barriers of particular pertinence to ethnic minority and gender-diverse Autistic people revealed a lack of data directly attributable to those groups. Therefore, their lived experiences were not sufficiently represented. Where data attributable to these groups was reported, the barriers described were largely consistent with our wider findings. Concerns about not receiving a diagnosis were amplified by a lack of trust in clinician expertise with ethnic minority and gender-diverse groups (Ardeleanu et al., 2024; Tien et al., 2025). These concerns are not unfounded; research consistently demonstrates that both ethnic minority and gender-diverse communities experience a lack of accessibility, fears of discrimination, and poorer satisfaction and outcomes in relation to mental health services (Finneran et al., 2024; Lowther-Payne et al., 2023). Concerningly, gender-diverse autistic adults are more likely to experience mental health issues compared to their cisgender peers (Simpson et al., 2024), whilst ethnic minority Autistic people often struggle to access mental health support despite expressed need (Ames et al., 2022). As mental health concerns are often the primary reason for seeking an adult Autism diagnosis (Jones et al., 2014), these barriers are likely particularly consequential for these groups.

The persistent underrepresentation of ethnic minority and gender-diverse Autistic people in research, as demonstrated in this review, is part of a larger issue. Research findings may be based on unrepresentative—i.e., white, cisgender, socioeconomically advantaged—samples, thus biasing diagnostic practices and entrenching inequities in access to diagnosis (Kalb et al., 2022; Keates & Waldock, 2025). The observed mistrust of diagnostic services is therefore a reasonable response to these systemic issues and highlights an urgent need for more inclusive research practices to serve underrepresented groups.

Where reported, rates of co-occurring conditions, specifically anxiety, depression, and ADHD, were high. This was expected, given the high rates of these conditions in Autistic adults (Au-Yeung et al., 2019; Jadav & Bal, 2022). However, only three qualitative studies provided the number of participants with ID (Huang et al. 2024a, b, Huang et al. 2024b; Finch et al. 2022), and some excluded them altogether. While some autistic participants believed that the presence of ID could facilitate an Autism diagnosis (Tien et al., 2025), it remains unknown how many people with ID are diagnosed late. This reflects broader practices in Autism research which have been criticised for excluding those with ID (Beck et al., 2025; Russell et al., 2019), which is concerning, as over half of Autistic people are estimated to have ID (Loomes et al., 2017).

### Limitations of the Included Studies

While studies were generally methodologically sound, many were not transparent about researcher positionality and influence and reasons for case inclusion, thus raising the possibility of unaddressed researcher influence.

Some groups were not represented in this review. Nearly all studies were conducted in Western, mostly anglophone countries. Our conclusions are therefore limited to those settings. Barriers to diagnostic or post-diagnostic services are different in other countries, and such services may not even be provided in some locations (Lewis, 2017).

As shown, very few studies explicitly represented ethnic minority or gender-diverse participants. Terms such as ‘sex’, ‘gender’, and ‘gender identity’ were sometimes used interchangeably, and as a result, some gender-diverse participants’ gender may not have been recorded appropriately.

Our findings are, most likely, limited by self-selection effects, echoing broader concerns about selective representation in autism research (Russell et al., 2019). Interviews privileged verbally fluent participants, whilst internet surveys are not accessible to many autistic people (Rubenstein & Furnier, 2021). Moreover, although autistic participants and family members reported a great variety of experiences, it is possible that those who experienced few barriers to diagnosis or placed little importance on it were less incentivised to participate in research. The perspectives of parents who did not support their adult children in seeking an autism diagnosis were also absent. Specialists who were better informed about late diagnosis might be more likely to partake in related studies. However, this review underscored that the experiences of many autistic adults with their clinicians were far from positive, which points at a gap in the availability of specialists or a mismatch between the clinicians’ and clients’ perceptions.

### Strengths and Limitations of this Review

A major contribution of this review is the inclusion of both quantitative and qualitative studies from the perspectives of three key stakeholder groups, which enabled us to identify and interpret areas of discordance. The broader definition of barriers as factors associated with a late diagnosis allowed us to report on a greater number of challenges.

TS always involves a degree of author subjectivity (Thomas & Harden, 2008), so a different group of authors would have likely generated different themes. We performed a GRADE-CERQual assessment of the findings to ensure rigour and coherence and report any concerns transparently. Most findings were judged as medium to high confidence, suggesting that the experiences presented here are representative of late-diagnosed and self-identifying autistic people without ID in the West.

We included the perspectives of self-identifying autistic people. Our goal was to maximise the fairness of the review due to the many barriers to diagnosis, which we explored in detail. We assumed that self-identification is made in good faith (Sarrett, 2016), and it indeed often precedes a late diagnosis (Lewis, 2017). However, a limitation of including self-identifying participants is that there is no way of knowing if autism would be an appropriate diagnosis for them.

### Implications and Future Directions

As our review highlighted that lived experience and clinician perspectives on autism diagnosis diverge significantly, the potential for shared decision-making (Edwards & Elwyn, 2006; Montori et al., 2023) models should be explored. Such approaches have shown promise in enhancing patient care in other areas of medicine (Stiggelbout et al., 2012). Considering our findings, their emphasis on exploring meaning-making around aspects of care and collaboratively reconciling disagreements, could benefit both service users and clinicians, thus resulting in more satisfying assessment and post-diagnostic experiences.

Another possibility is involving autistic clinicians in the diagnostic process. Emerging research on autistic psychiatrists suggests that could enhance recognition and rapport with autistic patients (Doherty et al., 2024). However, it is important to note that autistic-autistic communication is not universally seamless, as exemplified by the reviewed findings about tensions in autistic spaces (Huang et al. 2024a; Seers and Hogg 2023) as well as other research on the friction that can arise in autistic-autistic interactions (Botha et al., 2022; Khudiakova et al., 2025). Therefore, services must be sufficiently flexible and sensitive to individual communication needs.

Our review demonstrates that finding appropriately qualified and trustworthy specialists, as well as travelling to and attending appointments, may present significant barriers. Therefore, the potential for telehealth diagnostic services should be explored, in order to provide access to individuals who reside in areas lacking professionals with appropriate expertise or who struggle to attend in-person appointments. Online adaptations of Autism diagnostic tools have shown promise in some research, performing comparably to their original versions (e.g., Blackmore et al., 2023). However, such tools must be further validated on diverse samples, particularly in terms of sex, gender, and ethnicity, to ensure their inclusivity and sensitivity and avoid perpetuating diagnostic biases.

It is important for research to understand the experiences of ethnic minority and gender-diverse late adolescents and adults seeking an Autism diagnosis and whether they are disadvantaged by existing diagnostic tools and practices. Intersectional, participatory approaches would be beneficial in shedding light on those experiences and on how current systems entrench existing privileges and biases (Botha and Gillespie-Lynch 2022; Davies et al. 2024b). Additionally, large-scale studies should examine the prevalence rates of co-occurring conditions in late-diagnosed Autistic people to help identify those who may be at risk of remaining unrecognised. Finally, the experiences of late-diagnosed Autistic people with ID should be studied to understand if they face different barriers from those without ID.

### Clinical Implications

In response to the barriers reported in this review, we propose six recommendations for clinical practice.

1. Health services should ensure that information on available diagnostic services and referral processes is centralised and accessible.
2. Those who self-identify as Autistic should not be pressured into undergoing an assessment, especially if there is a risk of a loss of rights, but instead provided with individually tailored support.
3. Training resources, ideally case-based, should be created for referring professionals, including GPs. The training should incorporate identifying possible Autism in people who have received other mental health diagnoses.
4. Due to the importance of post-diagnostic care and support, previously accessed supports should not be withdrawn following diagnosis until there is an acceptable alternative secured.
5. Shared decision-making or community informed diagnostic models should be explored to reduce disadvantage.
6. If a diagnosis is not made, the clinician’s reasoning should be justified to the client, with an option to seek a second opinion. Next steps in terms of support needs should be explored.

## Conclusion

Accessing a late Autism diagnosis and post-diagnostic supports is often a challenging process. This review explored the perspectives of late-diagnosed or self-identifying autistic people, family members, and clinicians, revealing compounding barriers such as individual attitudes, mistrust of diagnostic tools and criteria, lack of clinician awareness, limited services, and financial and systemic barriers. There are significant areas of divergence between the three stakeholder groups, underscoring the importance of dialogue. Our findings also highlight the unique barriers faced by gender-diverse and ethnic minority Autistic people, although there was a notable lack of representation of these groups. More inclusive approaches are needed to make diagnostic and post-diagnostic services fairer and easier to access.

## Supplementary Information

Below is the link to the electronic supplementary material.

ESM 1 (DOCX 75.4 KB)

## Data Availability

No datasets were generated or analysed during the current study.

## Appendix A: Search Terms

PsychINFO, Medline, and Embase (via Ovid).

((autis* or asperger* or ASD or “autism spectrum disorder” or “autistic spectrum disorder” or ASC or “autism spectrum condition” or “autistic spectrum condition” or PDD or “pervasive developmental disorder”) and ((adult adj2 diagnos*) or adult-diagnos* or (late adj2 diagnos*) or (late adj2 identif*) or (adult adj2 identif*))).mp. [mp=title, abstract]

Web of Science.

TS=((autis* OR asperger* OR ASD OR “autism spectrum disorder” OR “autistic spectrum disorder” OR ASC OR “autism spectrum condition” OR “autistic spectrum condition” OR PDD OR “pervasive developmental disorder”) AND ((adult NEAR/2 diagnos*) OR adult-diagnos* OR (late NEAR/2 diagnos*) OR (late NEAR/2 identif*) OR (adult NEAR/2 identif*))).

ProQuest Dissertations & Theses.

(autis* or asperger* or ASD or “autism spectrum disorder” or “autistic spectrum disorder” or ASC or “autism spectrum condition” or “autistic spectrum condition” or PDD or “pervasive developmental disorder”) and((adult n2 diagnos*) or adult-diagnos* or (late n2 diagnos*) or (late n2 identif*) or (adult n2 identif*)).

## Appendix B: Study Characteristics

**Table 4:** Study characteristics

Author, Year	*N*	Methodology	Diagnostic Status	Mean Age	Gender/Sex	Country	Quality Score
Ardeleanu et al., 2024	65	Qualitative interviews	27 LDX, 10 CDX, 28 SID	29	10 M, 24 F, 31 O	USA	8/10^a^
Atherton et al., 2022 (Study 1)	399	Quantitative cross-sectional	98 LDX, 101 CDX, 191 NA	25.53	179 M, 220 F	UK	4/8^b^
Atherton et al., 2022 (Study 2)	8	Qualitative interviews	8 LDX	39.88	4 M, 4 F	UK	8/10^a^
Bargiela et al., 2016	14	Qualitative interviews	14LDX	26.7	14 F	UK	8/10^a^
Bakken & Høidal, 2014	12	Chart analysis	4 LDX, 1 NA, 7 unknown	36.5^*^	10 M, 2 F	Norway	8/9^c^
Bradley et al., 2021	277	Qualitative survey	206 LDX, 71 SID	38.59	93 M, 184 F	UK	7/10^a^
Cage et al., 2024	6	Qualitative interviews	6 SID	32.67	6 F	UK	10/10^a^
Castañeda, 2023	8	Qualitative interviews	8 LDX	49^a^	8 F	USA	8/10^a^
Craddock, 2024	6	Qualitative interviews	6 LDX	44.5^a^	6 F	UK	10/10^a^
Cuda Pierce, 2022	15	Qualitative interviews	2 LDX, 13 SID	35.67	3 M, 9 F, 3O	USA	6/10^a^
Cumin et al., 2022	20	Delphi study	20 CL	NR	NR	International	Moderate^d^
Darling Rasmussen, 2023	6	Case series	6 LDX	29.83	0 M, 6 F	Denmark	4/9^c^
de Broize et al., 2022	13	Qualitative interviews	10 LDX, 3 SID	51.2	5 M, 8 F	Australia	10/10^a^
Dodd, 2022 (Study 1)	201	Quantitative survey	71 LDX, 29 CDX, 101 SID	36.63	59 M, 100 F, 39O	International	6/8^b^
Dodd, 2022 (Study 2)	208	Quantitative survey	103 LDX, 33 CDX, 72 SID	35.39	36 M, 149 F, 23O	UK	6/8^b^
Dubreucq et al., 2023	383	Chart analysis	383 LDX	28.1	262 M, 121 F	France	8/8^b^
Evans, 2022	9	Qualitative interviews	9 LDX	35^*^	Not reported	UK	10/10^a^
Finch et al., 2022	45	Qualitative interviews	27 LDX, 2 CDX, 16 FM	47.00 (autistic), 57.57 (family)	22 M, 23 F (autistic), 3 M, 13 F (family)	UK	7/10^a^
Fiori Nastro et al., 2023	1	Case study	1 LDX	19	1 M	Italy	5/5^e^
Freeman & Paradis, 2023	8	Qualitative interviews	8 FM	Not reported	8 F	Australia	8/10^a^
Friedman et al., 2024	6	Qualitative interviews	6 SID	34.67	4 F, 2O	UK	7/10^a^
Gerhand, 2020	8	Qualitative interviews	8 LDX	53.8	3 M, 5 F	UK	7/10^a^
Ghanouni & Seaker, 2023	18	Qualitative interviews	13 LDX, 5FM	40 (autistic), 58 (family)	9 M, 4 F (autistic), 2 M, 3 F (family)	Canada	7/10^a^
Gilmore & Hayes, 1996	1	Case study	1 LDX	17	1 M	Australia	4/5^e^
Gonçalves Garcia et al., 2025	253	Quantitative survey	161 LDX, 92SID	33.1	66 M, 171 F, 16O	Brazil	6/8^b^
Jadav & Bal, 2022	4657	Quantitative survey	2437 CDX, 2220 LDX	33.4	2343 M, 2314 F	USA	8/8^b^
Hamdani et al., 2023	37	Qualitative interviews & focus groups	18 LDX, 4 CDX, 15 FM	41.5^*^	37 F	Canada	8/10^a^
Hayes et al., 2021	11	Conversation analysis	11 LDX	36.8^*^	4 M, 7 F	UK	7/10^a^
Heijnen-Kohl et al., 2025	11	Delphi study	11 CL	Not reported	Not reported	Netherlands	Moderate^d^
Huang et al. 2024a	23	Qualitative interviews	19 LDX, 4 FM	44.37 (adults), 52.5^a^ (family)	7 M, 16 F	Australia	10/10^a^
Huang et al. 2024b	137	Qualitative survey	137 LDX	41.91	34 M, 86 F, 14O	Australia	10/10^a^
Lam et al., 2024	13	Qualitative interviews	5 LDX, 2 CDX, 6 SID	33.43	13 F	Hong Kong	10/10^a^
Leedham et al., 2020	11	Qualitative interviews	11 LDX	50.81	11 F	UK	8/10^a^
Legg et al., 2023	11	Qualitative interviews	11FM	Not reported	2 M, 9 F	UK	8/10^a^
Lewis, 2017 (Qual)	114	Qualitative survey	60 LDX, 54 SID	36.2	62 M, 48 F, 4O	International	7/10^a^
Lewis, 2017 (Quant)	665	Quantitative cross-sectional	227 LDX, 437 SID	30.9	94 M, 381 F, 190O	International	4/8^b^
Lupindo et al., 2023	10	Qualitative interviews	10 LDX	42.70	10 M, 0 F	South Africa	10/10^a^
McKenzie et al., 2015	70	Quantitative cross-sectional	70 LDX	31.7	44 M, 24 F, 2O	UK	7/8^d^
McQuaid et al., 2022	502	Quantitative cross-sectional	251 CDX, 251 LDX	32.97	225 M, 277 F	USA	8/8^b^
Murphy et al., 2023	10	Qualitative interviews	10 LDX	40.60	10 F	Australia	10/10^a^
Pearse, 2020	5	Qualitative interviews	4 LDX, 1 SID	39^*^	5 F	UK	6/10^a^
Powell & Acker, 2016	74	Qualitative survey	54 LDX, 20 NA	36.08	44 M, 30 F	UK	7/10^a^
Punshon et al., 2009	10	Qualitative interviews	10 LDX	31 (median)	7 M, 3 F	UK	6/10^a^
Raymond-Barker et al., 2018	6	Qualitative interviews	6 FM	56.50	6 F	UK	7/10^a^
Roy & Balaratnasingam, 2010	14	Case series	13 LDX, 1 non-diagnosed	31.36	9 M, 5 F	Australia	7/9^c^
Scattoni et al., 2021	595	Online survey	356 LDX, 88 FM, 151 CL	38.25^*^ (autistic), 54.00^*^ (family)	151 M, 430 F, 14O	International	5/8^b^
Seers & Hogg, 2023	8	Qualitative interviews	8 LDX	39.12	8 F	Australia	8/10^a^
Smith, 2021	3	Case series	3 LDX	35^*^	3 M	UK	4/10^c^
Stagg & Belcher, 2019	9	Qualitative interviews	9 LDX	53^*^	4 M, 5 F	UK	10/10^a^
Tamilson et al., 2025	10	Qualitative interviews	10 LDX	34	0 M, 9 F, 1O	UK	10/10^a^
Tien et al., 2025	24	Qualitative interviews	15 LDX, 1 CDX, 8 SID	26.13	2 M, 8 F, 14O	USA	8/10^a^
van Niekerk et al., 2011	3	Case series	3 LDX	77.67	3 M	Netherlands	5/9^c^
Wigham et al., 2022 and Wigham et al., 2023	423	Delphi study	343 LDX, 45FM, 35CL	43.2 (autistic), not reported for other groups	169 M, 239 F (autistic), not reported for other groups	UK	4/8^b^

LDX = late diagnosed, CDX = child diagnosed, SID = self-identifying, NA = non-autistic, FM = family members, CL = clinicians. Detailed data on ethnicity, where recorded, is provided in the Supplement (Table S.1). Co-occurring conditions, where reported, are summarised in the Supplement (Table S.2)

^a^Joanna Briggs Checklist for Qualitative Research (Lockwood et al., 2015); ^b^Joanna Briggs Checklist for Analytical Cross-Sectional Studies (Moola et al. 2020); ^c^Joanna Briggs Checklist for Case Series (Munn, et al., 2020);^d^Delphi Critical Appraisal Checklist (Grant, and Khodyakov, 2025); ^e^Joanna Briggs Checklist for Case Reports (Moola et al., 2017)

* Estimated via calculating the midpoint of the age range

## Footnotes

1 To respect the preferences of some Autistic people and communities and emphasise shared Autistic community identity, we capitalise ‘Autistic’ and ‘Autism’ (Benham and Kizer 2016; Davies et al. 2024a).

2 Additional barriers posed by previous and co-occurring diagnoses are explored in Subtheme 3.1.

3 Note that gender identity and sex assigned at birth were not always specified by studies.

4 It is noteworthy that the Delphi study by Heijnen-Kohl et al. (2025) did not provide an age range for the population described as ‘older adults’, while the case series by van Niekerk et al. (2011) included individuals ranging from 72 to 84 years of age.

## Declarations

### Data Sharing

Data sharing is not applicable as this review relies solely on previously published research.
