# Supplementary material for: Lived Experience, Family, and Clinician Perspectives on Barriers to Adult Autism Diagnosis and Post-Diagnostic Supports: A Mixed-Methods Systematic Review
Source: J Dev Phys Disabil. 2026 Mar 5;38(5):979–1018. doi: 10.1007/s10882-026-10055-x (PMC13615933; doi:10.1007/s10882-026-10055-x)
Supplement: Supplementary file 1 [file 10882_2026_10055_MOESM1_ESM.docx]

Supplementary Materials

**PRISMA 2020 Main Checklist**

| Topic | No. | Item | Location where item is reported |
| --- | --- | --- | --- |
| TITLE |  |  |  |
| Title | 1 | Identify the report as a systematic review. | Title |
| ABSTRACT |  |  |  |
| Abstract | 2 | See the PRISMA 2020 for Abstracts checklist |  |
| INTRODUCTION |  |  |  |
| Rationale | 3 | Describe the rationale for the review in the context of existing knowledge. | Rationale |
| Objectives | 4 | Provide an explicit statement of the objective(s) or question(s) the review addresses. | Rationale |
| METHODS |  |  |  |
| Eligibility criteria | 5 | Specify the inclusion and exclusion criteria for the review and how studies were grouped for the syntheses. | Methods |
| Information sources | 6 | Specify all databases, registers, websites, organisations, reference lists and other sources searched or consulted to identify studies. Specify the date when each source was last searched or consulted. | Methods |
| Search strategy | 7 | Present the full search strategies for all databases, registers and websites, including any filters and limits used. | Methods, Appendix |
| Selection process | 8 | Specify the methods used to decide whether a study met the inclusion criteria of the review, including how many reviewers screened each record and each report retrieved, whether they worked independently, and if applicable, details of automation tools used in the process. | Methods |
| Data collection process | 9 | Specify the methods used to collect data from reports, including how many reviewers collected data from each report, whether they worked independently, any processes for obtaining or confirming data from study investigators, and if applicable, details of automation tools used in the process. | Methods |
| Data items | 10a | List and define all outcomes for which data were sought. Specify whether all results that were compatible with each outcome domain in each study were sought (e.g. for all measures, time points, analyses), and if not, the methods used to decide which results to collect. | Methods |
|  | 10b | List and define all other variables for which data were sought (e.g. participant and intervention characteristics, funding sources). Describe any assumptions made about any missing or unclear information. | Methods |
| Study risk of bias assessment | 11 | Specify the methods used to assess risk of bias in the included studies, including details of the tool(s) used, how many reviewers assessed each study and whether they worked independently, and if applicable, details of automation tools used in the process. | Methods |
| Effect measures | 12 | Specify for each outcome the effect measure(s) (e.g. risk ratio, mean difference) used in the synthesis or presentation of results. | NA (quotes) |
| Synthesis methods | 13a | Describe the processes used to decide which studies were eligible for each synthesis (e.g. tabulating the study intervention characteristics and comparing against the planned groups for each synthesis (item 5)). | Methods |
|  | 13b | Describe any methods required to prepare the data for presentation or synthesis, such as handling of missing summary statistics, or data conversions. | N/A |
|  | 13c | Describe any methods used to tabulate or visually display results of individual studies and syntheses. | Supplement |
|  | 13d | Describe any methods used to synthesize results and provide a rationale for the choice(s). If meta-analysis was performed, describe the model(s), method(s) to identify the presence and extent of statistical heterogeneity, and software package(s) used. | Methods |
|  | 13e | Describe any methods used to explore possible causes of heterogeneity among study results (e.g. subgroup analysis, meta-regression). | Methods (Objectives 2 and 3) |
|  | 13f | Describe any sensitivity analyses conducted to assess robustness of the synthesized results. | N/A |
| Reporting bias assessment | 14 | Describe any methods used to assess risk of bias due to missing results in a synthesis (arising from reporting biases). | N/A |
| Certainty assessment | 15 | Describe any methods used to assess certainty (or confidence) in the body of evidence for an outcome. | Methods, Supplement |
| RESULTS |  |  |  |
| Study selection | 16a | Describe the results of the search and selection process, from the number of records identified in the search to the number of studies included in the review, ideally using a flow diagram. | Results |
|  | 16b | Cite studies that might appear to meet the inclusion criteria, but which were excluded, and explain why they were excluded. | Supplement |
| Study characteristics | 17 | Cite each included study and present its characteristics. | Results |
| Risk of bias in studies | 18 | Present assessments of risk of bias for each included study. | Supplement |
| Results of individual studies | 19 | For all outcomes, present, for each study: (a) summary statistics for each group (where appropriate) and (b) an effect estimate and its precision (e.g. confidence/credible interval), ideally using structured tables or plots. | N/A |
| Results of syntheses | 20a | For each synthesis, briefly summarise the characteristics and risk of bias among contributing studies. | Results |
|  | 20b | Present results of all statistical syntheses conducted. If meta-analysis was done, present for each the summary estimate and its precision (e.g. confidence/credible interval) and measures of statistical heterogeneity. If comparing groups, describe the direction of the effect. | N/A |
|  | 20c | Present results of all investigations of possible causes of heterogeneity among study results. | Results (Objectives 2 and 3) |
|  | 20d | Present results of all sensitivity analyses conducted to assess the robustness of the synthesized results. | Results (Objectives 2 and 3) |
| Reporting biases | 21 | Present assessments of risk of bias due to missing results (arising from reporting biases) for each synthesis assessed. | N/A |
| Certainty of evidence | 22 | Present assessments of certainty (or confidence) in the body of evidence for each outcome assessed. | Results, Supplement |
| DISCUSSION |  |  |  |
| Discussion | 23a | Provide a general interpretation of the results in the context of other evidence. | Discussion |
|  | 23b | Discuss any limitations of the evidence included in the review. | Discussion |
|  | 23c | Discuss any limitations of the review processes used. | Discussion |
|  | 23d | Discuss implications of the results for practice, policy, and future research. | Discussion |
| OTHER INFORMATION |  |  |  |
| Registration and protocol | 24a | Provide registration information for the review, including register name and registration number, or state that the review was not registered. | Methods |
|  | 24b | Indicate where the review protocol can be accessed, or state that a protocol was not prepared. | Methods |
|  | 24c | Describe and explain any amendments to information provided at registration or in the protocol. | Methods |
| Support | 25 | Describe sources of financial or non-financial support for the review, and the role of the funders or sponsors in the review. | Acknowledgements |
| Competing interests | 26 | Declare any competing interests of review authors. | Acknowledgements |
| Availability of data, code and other materials | 27 | Report which of the following are publicly available and where they can be found: template data collection forms; data extracted from included studies; data used for all analyses; analytic code; any other materials used in the review. | Supplement |

PRISMA Abstract Checklist

| Topic | No. | Item | Reported? |
| --- | --- | --- | --- |
| TITLE |  |  |  |
| Title | 1 | Identify the report as a systematic review. | Yes |
| BACKGROUND |  |  |  |
| Objectives | 2 | Provide an explicit statement of the main objective(s) or question(s) the review addresses. | Yes |
| METHODS |  |  |  |
| Eligibility criteria | 3 | Specify the inclusion and exclusion criteria for the review. | Yes |
| Information sources | 4 | Specify the information sources (e.g. databases, registers) used to identify studies and the date when each was last searched. | Yes |
| Risk of bias | 5 | Specify the methods used to assess risk of bias in the included studies. | Yes |
| Synthesis of results | 6 | Specify the methods used to present and synthesize results. | Yes |
| RESULTS |  |  |  |
| Included studies | 7 | Give the total number of included studies and participants and summarise relevant characteristics of studies. | Yes |
| Synthesis of results | 8 | Present results for main outcomes, preferably indicating the number of included studies and participants for each. If meta-analysis was done, report the summary estimate and confidence/credible interval. If comparing groups, indicate the direction of the effect (i.e. which group is favoured). | Yes |
| DISCUSSION |  |  |  |
| Limitations of evidence | 9 | Provide a brief summary of the limitations of the evidence included in the review (e.g. study risk of bias, inconsistency and imprecision). | Yes |
| Interpretation | 10 | Provide a general interpretation of the results and important implications. | Yes |
| OTHER |  |  |  |
| Funding | 11 | Specify the primary source of funding for the review. | Yes |
| Registration | 12 | Provide the register name and registration number. | Yes |

*From:* Page MJ, McKenzie JE, Bossuyt PM, Boutron I, Hoffmann TC, Mulrow CD, et al. The PRISMA 2020 statement: an updated guideline for reporting systematic reviews. MetaArXiv. 2020, September 14. DOI: 10.31222/osf.io/v7gm2. For more information, visit: **www.prisma-statement.org**

**ENTREQ Checklist**

|  | **Item** | **Clarification** | **Location** |
| --- | --- | --- | --- |
| 1 | Aim | State the research question the synthesis addresses. | Rationale |
| 2 | Synthesis methodology | Identify the synthesis methodology or theoretical framework which underpins the synthesis, and describe the rationale for choice of methodology (e.g. meta-ethnography, thematic synthesis, critical interpretive synthesis, grounded theory synthesis, realist synthesis, meta-aggregation, meta-study, framework synthesis). | Methods |
| 3 | Approach to searching | Indicate whether the search was pre-planned (comprehensive search strategies to seek all available studies) or iterative (to seek all available concepts until they theoretical saturation is achieved). | Methods |
| 4 | Inclusion criteria | Specify the inclusion/exclusion criteria (e.g. in terms of population, language, year limits, type of publication, study type). | Methods |
| 5 | Data sources | Describe the information sources used (e.g. electronic databases (MEDLINE, EMBASE, CINAHL, psycINFO, Econlit), grey literature databases (digital thesis, policy reports), relevant organisational websites, experts, information specialists, generic web searches (Google Scholar) hand searching, reference lists) and when the searches conducted; provide the rationale for using the data sources. | Methods |
| 6 | Electronic Search strategy | Describe the literature search (e.g. provide electronic search strategies with population terms, clinical or health topic terms, experiential or social phenomena related terms, filters for qualitative research, and search limits). | Methods, Appendix |
| 7 | Study screening methods | Describe the process of study screening and sifting (e.g. title, abstract and full text review, number of independent reviewers who screened studies). | Methods |
| 8 | Study characteristics | Present the characteristics of the included studies (e.g. year of publication, country, population, number of participants, data collection, methodology, analysis, research questions). | Results |
| 9 | Study selection results | Identify the number of studies screened and provide reasons for study exclusion (e.g. for comprehensive searching, provide numbers of studies screened and reasons for exclusion indicated in a figure/flowchart; for iterative searching describe reasons for study exclusion and inclusion based on modifications to the research question and/or contribution to theory development). | Results |
| 10 | Rationale for appraisal | Describe the rationale and approach used to appraise the included studies or selected findings (e.g. assessment of conduct (validity and robustness), assessment of reporting (transparency), assessment of content and utility of the findings). | Methods |
| 11 | Appraisal items | State the tools, frameworks and criteria used to appraise the studies or selected findings (e.g. Existing tools: CASP, QARI, COREQ, Mays and Pope[25]; reviewer developed tools; describe the domains assessed: research team, study design, data analysis and interpretations, reporting). | Methods |
| 12 | Appraisal process | Indicate whether the appraisal was conducted independently by more than one reviewer and if consensus was required. | Result |
| 13 | Appraisal results | Present results of the quality assessment and indicate which articles, if any, were weighted/excluded based on the assessment and give the rationale. | Result |
| 14 | Data extraction | Indicate which sections of the primary studies were analysed and how were the data extracted from the primary studies? (e.g. all text under the headings "results /conclusions" were extracted electronically and entered into a computer software). | Methods |
| 15 | Software | State the computer software used, if any. | Methods |
| 16 | Number of reviewers | Identify who was involved in coding and analysis. | Methods |
| 17 | Coding | Describe the process for coding of data (e.g. line by line coding to search for concepts). | Methods |
| 18 | Study comparison | Describe how were comparisons made within and across studies (e.g. subsequent studies were coded into pre-existing concepts, and new concepts were created when deemed necessary). | Methods |
| 19 | Derivation of themes | Explain whether the process of deriving the themes or constructs was inductive or deductive. | Methods |
| 20 | Quotations | Provide quotations from the primary studies to illustrate themes/constructs, and identify whether the quotations were participant quotations of the author's interpretation. | Results |
| 21 | Synthesis output | Present rich, compelling and useful results that go beyond a summary of the primary studies (e.g. new interpretation, models of evidence, conceptual models, analytical framework, development of a new theory or construct). | Results, Discussion |

Examples of Excluded Studies

Jones, L., Goddard, L., Hill, E. L., Henry, L. A., & Crane, L. (2014). Experiences of receiving a diagnosis of autism spectrum disorder: A survey of adults in the United Kingdom. *Journal of Autism and Developmental Disorders, 44*(12), 3033–3044. doi:10.1007/s10803-014-2161-3

**Reason for exclusion:** Data reported together on child- and adult-diagnosed participants

Powell, T., Parker, J., Kitson, H., & Rogalewski, M. (2024). “It Was Like the Final Piece in the Puzzle for Me”: A Qualitative Study on the Experiences of Autistic Women Initially Diagnosed with Borderline Personality Disorder. *Autism in Adulthood*, *6*(4), 428-437.

**Reason for exclusion:** Does not discuss how a diagnosis of BPD may be a barrier to autism diagnosis, or any other barriers.

Detailed Study Demographics

Table S.1

*Ethnic Composition of Studies, Where Available*

| Study | N | % White | % Black | % Asian | % Indigenous | % Other |
| --- | --- | --- | --- | --- | --- | --- |
| Ardeleanu et al. (2024) | 65^a^ | 68 | NR | NR | NR | 25 ‘of colour’. 7 unknown |
| Atherton et al., 2022 (Study 2) | 8 | 100 | 0 | 0 | 0 | 0 |
| Cage et al., 2024 | 6 | 100 | 0 | 0 | 0 | 0 |
| Castañeda, 2023 | 8 | 75 | 0 | 0 | 0 | 25 Hispanic |
| Cuda Pierce, 2022 | 15 | 60 | 27 | 0 | 0 | 13 |
| de Broize et al., 2022 | 13 | 85 | 0 | 15 | 0 | 0 |
| Huang et al., 2024b | 137 | 90 | NR | NR | NR | 10 non-white |
| Lam et al., 2024 | 13 | 0 | 0 | 100 | 0 | 0 |
| Lewis, 2017, quantitative | 634 | 87 | 2 | 2 | <1 | 3 Hispanic, 6 Mixed race |
| Lewis, 2017, qualitative | 114 | 88 | 4 | 4 | 2 | 2 |
| Lupindo et al., 2023 | 10 | 100 | 0 | 0 | 0 | 0 |
| McQuaid et al., 2022 | 251 | 82 | 2 | 2 | <1 | 10 more than one race, 3 other |
| Pearse, 2020 | 5 | 100 | 0 | 0 | 0 | 0 |
| Tien et al., 2025 | 24 | 79 | 0 | 4 | 0 | 17 |

^a^ Includes four childhood-diagnosed participants due to aggregate reporting.

^b^ Includes the 20 subclinical participants due to aggregate reporting.

**Table S.2**

*Co-Occurring Conditions Reported in Studies*

| Condition | Finch  (n = 27)^a^ | Seers & Hogg (n = 8) | Lupindo (n = 10) | Bradley  (n =206) ^a^ | Tamilson  (n = 10) | Craddock (n = 6) | Huang  2024a  (n = 19) | Huang 2024b (n = 137) | Jadav & Bal (n = 2210)^a^ |
| --- | --- | --- | --- | --- | --- | --- | --- | --- | --- |
| % Any | 89 | 50 | 80 | 89 | 100 | 100 | 21^b^ | 91 | 91 |
| % Depression | 70 | 38 | 10 | 79 | - | - | - | 84 | 61 |
| % Anxiety | 56 | 38 | - | 70 | - | - | - | 82 | 60 |
| % Bipolar disorder | 19 | - | - | 7 | - | - | - | - | 16 |
| % ADHD | 145 | 25 | 30 | - | - | 100 | - | 42 | 42 |
| % OCD | 11 | 13 | - | 19 | - | - | - | - | 25 |
| % ID | 4 | - | - | - | - | - | 21 | 6 | - |
| % Dyslexia | 7 | - | 20 | - | - | - | - | - | - |
| % Dyspraxia | 7 | - | 10 | - | - | - | - | - | - |
| % Eating disorders | - | 13 | - | 8 | - | - | - | - | 12 |
| % Social anxiety | - | 13 | - | - | - | - | - | - | 41 |
| % PTSD | - | - | 10 | - | - | - | - | 30 | - |
| % Learning difficulties | - | - | 10 | - | - | - | - | - | - |
| % Personality disorder | - | - | - | 12 | 100 | - | - | - | 15 |
| % Schizophrenia | - | - | - | 4 | - | - | - | - | 4 |

^a^ Late-diagnosed participants only.

^b^ ID was the only condition reported.

Summary of Quality Assessment Results

Table S.3

*Joanna Briggs Checklist for Qualitative Research*

| First Author | 1 | 2 | 3 | 4 | 5 | 6 | 7 | 8 | 9 | 10 | Total |
| --- | --- | --- | --- | --- | --- | --- | --- | --- | --- | --- | --- |
| Ardeleanu | Y | Y | Y | Y | Y | N | N | Y | Y | Y | 8 |
| Atherton (Study 2) | Y | Y | Y | Y | Y | N | N | Y | Y | Y | 8 |
| Bargiela | Y | Y | Y | Y | Y | N | N | Y | Y | Y | 8 |
| Bradley | Unclear | Y | Y | Y | Y | N | Unclear | Y | Y | Y | 7 |
| Cage | Y | Y | Y | Y | Y | Y | Y | Y | Y | Y | 10 |
| Castañeda | Unclear | Y | Y | Y | Y | Y | Unclear | Y | Y | Y | 8 |
| Craddock | Y | Y | Y | Y | Y | Y | Y | Y | Y | Y | 10 |
| Cuda Pierce | Y | Y | Y | Unclear | Y | N | Unclear | Unclear | Y | Y | 6 |
| de Broize | Y | Y | Y | Y | Y | Y | Y | Y | Y | Y | 10 |
| Evans | Y | Y | Y | Y | Y | Y | Y | Y | Y | Y | 10 |
| Finch | Y | Y | Y | Y | Unclear | N | N | Y | Y | Y | 7 |
| Freeman | Y | Y | Y | Y | Y | N | Unclear | Y | Y | Y | 8 |
| Friedman | Unclear | Y | Y | Y | Y | Y | Y | Y | Y | Y | 9 |
| Gerhand | Unclear | Y | Y | Y | Y | N | Unclear | Y | Y | Y | 7 |
| Ghanouni | Unclear | Y | Y | Y | Y | N | Unclear | Y | Y | Y | 7 |
| Hamdani | Y | Y | Y | Y | Y | N | N | Y | Y | Y | 8 |
| Hayes | Unclear | Y | Y | Y | Y | N | N | Y | Y | Y | 7 |
| Huang 2022a | Y | Y | Y | Y | Y | Y | Y | Y | Y | Y | 10 |
| Huang 2022b | Y | Y | Y | Y | Y | Y | Y | Y | Y | Y | 10 |
| Lam | Y | Y | Y | Y | Y | Y | Y | Y | Y | Y | 10 |
| Leedham | Y | Y | Y | Y | Y | N | N | Y | Y | Y | 8 |
| Legg | Y | Y | Y | Y | Y | N | Unclear | Y | Y | Y | 8 |
| Lewis (qualitative) | Unclear | Y | Y | Y | Y | N | N | Y | Y | Y | 7 |
| Lupindo | Y | Y | Y | Y | Y | Y | Y | Y | Y | Y | 10 |
| Murphy | Y | Y | Y | Y | Y | Y | Y | Y | Y | Y | 10 |
| Pearse | Y | Y | Y | Y | Y | Y | Y | Y | Y | Y | 10 |
| Powell | Unclear | Y | Y | Y | Y | N | N | Y | Y | Y | 6 |
| Punshon | Unclear | Y | Y | Y | Unclear | N | N | Y | Y | Y | 6 |
| Raymond-Barker | Unclear | Y | Y | Y | Y | N | N | Y | Y | Y | 7 |
| Seers | Y | Y | Y | Y | Y | N | N | Y | Y | Y | 8 |
| Stagg | Y | Y | Y | Y | Y | Y | Y | Y | Y | Y | 10 |
| Tamilson | Y | Y | Y | Y | Y | Y | Y | Y | Y | Y | 10 |
| Tien | Y | Unclear | Y | Y | Unclear | Y | Y | Y | Y | Y | 8 |

Checklist items (Lockwood et al., 2015):

1. Is there congruity between the stated philosophical perspective and the research methodology?
2. Is there congruity between the research methodology and the research question or objectives?
3. Is there congruity between the research methodology and the methods used to collect data?
4. Is there congruity between the research methodology and the representation and analysis of data?
5. Is there congruity between the research methodology and the interpretation of results?
6. Is there a statement locating the researcher culturally or theoretically?
7. Is the influence of the researcher on the research, and vice-versa, addressed?
8. Are participants, and their voices, adequately represented?
9. Is the research ethical according to current criteria or, for recent studies, and is there evidence of ethical approval by an appropriate body?
10. Do the conclusions drawn in the research report flow from the analysis, or interpretation, of the data?

Table S.4

*Joanna Briggs Checklist for Case Series*

| First Author | 1 | 2 | 3 | 4 | 5 | 6 | 7 | 8 | 9 | 10 | Total (out of 9) |
| --- | --- | --- | --- | --- | --- | --- | --- | --- | --- | --- | --- |
| Bakken | Y | Y | Y | Unclear | Y | N | Y | Y | Unclear | N/A | 8 |
| Darling Rasmussen | Y | Unclear | Y | Unclear | Unclear | Unclear | Y | Y | N | N/A | 4 |
| Roy | Y | Y | Y | Unclear | Y | Y | Y | Unclear | Y | N/A | 7 |
| Smith | Unclear | Unclear | Y | Unclear | Unclear | Y | Y | Y | N | N/A | 4 |
| van Niekerk | Unclear | Y | Y | Unclear | Unclear | Y | Y | N | Y | N/A | 5 |

Checklist items (Munn et al., 2020)

1. Were there clear criteria for inclusion in the case series?
2. Was the condition measured in a standard, reliable way for all participants included in the case series?
3. Were valid methods used for identification of the condition for all participants included in the case series?
4. Did the case series have consecutive inclusion of participants?
5. Did the case series have complete inclusion of participants?
6. Was there clear reporting of the demographics of the participants in the study?
7. Was there clear reporting of clinical information of the participants?
8. Were the outcomes or follow up results of cases clearly reported?
9. Was there clear reporting of the presenting site(s)/clinic(s) demographic information?
10. Was statistical analysis appropriate?

Table S.5

*Joanna Briggs Checklist for Case Reports*

| **First Author** | 1 | 2 | 3 | 4 | 5 | 6 | 7 | 8 | Total (out of 5) |
| --- | --- | --- | --- | --- | --- | --- | --- | --- | --- |
| Fiori Nastro | Y | Y | Y | Y | N/A | N/A | N/A | Y | 5 |
| Gilmore | Y | Y | Y | N | N/A | N/A | N/A | Y | 4 |

Checklist items (Moola et al., 2017):

1. Were patient’s demographic characteristics clearly described?
2. Was the patient’s history clearly described and presented as a timeline?
3. Was the current clinical condition of the patient on presentation clearly described?
4. Were diagnostic tests or assessment methods and the results clearly described?
5. Was the intervention(s) or treatment procedure(s) clearly described?
6. Was the post-intervention clinical condition clearly described?
7. Were adverse events (harms) or unanticipated events identified and described?
8. Does the case report provide takeaway lessons?

Table S.6

*Joanna Briggs Checklist for Analytical Cross-Sectional Studies*

| Author | 1 | 2 | 3 | 4 | 5 | 6 | 7 | 8 | Total (out of 8) |
| --- | --- | --- | --- | --- | --- | --- | --- | --- | --- |
| Atherton (Study 1) | Y | Y | Unclear | Unclear | Unclear | Unclear | Y | Y | 4 |
| Dodd | Y | Y | Unclear* | N* | Y | Y | Y | Y | 6 |
| Dubreucq | Y | Y | Y | Y | Y | Y | Y | Y | 8 |
| Gonçalves Garcia | Y | Y | Unclear* | N* | Y | Y | Y | Y | 6 |
| Jadav | Y | Y | Y | Y | Y | Y | Y | Y | 8 |
| Lewis (quantitative) | Unclear | Y | Unclear* | N* | Y | Y | Unclear | Y | 4 |
| McKenzie | Y | Unclear | Y | Y | Y | Y | Y | Y | 7 |
| McQuaid | Y | Y | Y | Y | Y | Y | Y | Y | 8 |
| Scattoni | Y | Y | Unclear | Y | Unclear | Unclear | Y | Y | 5 |
| Wigham 2022 | Y | Y | Unclear | Unclear | Unclear | Unclear | Y | Y | 4 |
| Wigham 2023 | Y | Y | Unclear | Unclear | Unclear | Unclear | Y | Y | 4 |

***Self-identifying participants were included in line with the study objectives.

Checklist items (Moola et al., 2020):

1. Were the criteria for inclusion in the sample clearly defined?
2. Were the study subjects and the setting described in detail?
3. Was the exposure measured in a valid and reliable way?
4. Were objective, standard criteria used for measurement of the condition?
5. Were confounding factors identified?
6. Were strategies to deal with confounding factors stated?
7. Were the outcomes measured in a valid and reliable way?
8. Was appropriate statistical analysis used?

**Table S.7**

*Delphi Critical Appraisal Tool*

| Author | 1 | 2 | 3 | 4 | 5 | 6 | 7 | 8 | 9 | 10 | 11 | 12 | 13 | 14 | 15 | 16 | Overall quality |
| --- | --- | --- | --- | --- | --- | --- | --- | --- | --- | --- | --- | --- | --- | --- | --- | --- | --- |
| Cumin | Unclear | Y | Y | N | Y | Y | Y | N | Y | Y | Y | N | Y | Y | Y | Y | Moderate |
| Heijnen-Kohl | Unclear | Y | Y | N | Y | Y | Y | Y | Y | Y | Y | Y | N | Y | Y | Y | Moderate |

Checklist items (Grant & Khodyakov, 2025):

Core:

1. Anonymity: Did the research team appropriately employ anonymity in the Delphi study?
2. Iteration: Did the research team appropriately employ iteration in the Delphi study?
3. Statistical summary: Did the research team appropriately employ statistical summaries of group responses in the Delphi study?
4. Controlled feedback: Did the research team appropriately employ controlled feedback in the Delphi study?

Additional:

5. Justification for Delphi: Did the research team provide an appropriate justification for the use of the Delphi method in this study?
6. Type of Delphi: Did the research team provide an appropriate justification for the type of Delphi used in this study?
7. Panelist expertise: Did the research team seek the appropriate expertise for the Delphi study?
8. Panelist retention: Did the research team sufficiently retain panelists throughout the Delphi study?
9. Preliminary work: Did the research team conduct preliminary work before the first round of the Delphi study?
10. Design choices: Did the research team make appropriate design choices for the Delphi study?
11. Survey questions: Did the research team ask appropriate questions in the Delphi study?
12. Response items: Did the research team use appropriate response items in the Delphi study?
13. Pilot test: Did the research team conduct a pilot test before launching the Delphi study?
14. Data analysis: Did the research team appropriately analyze the data from the Delphi study?
15. Transparent reporting: Did the research team transparently report their Delphi study?
16. Ethical issues: Did the research team appropriately consider ethical issues?

Descriptive Themes

**Barriers to Diagnosis and Post-Diagnostic Services**

1. Lack of awareness and information (e.g., lack of clinician awareness, inaccessible information about local diagnostic and post-diagnostic services)
2. Stereotypes or misattributions (e.g., misconceptions held by clients, families and clinicians, diagnostic overshadowing)
3. Affective and attitudinal barriers (e.g., perceiving a clinical diagnosis as unduly pathologising, stigma attached to seeking help or neurodiversity, mistrust of services)
4. Inequities in the assessment process (e.g., perceived bias in tools, camouflaging during the assessment)
5. Fear of consequences (e.g., fear of stigma and discrimination)
6. Challenges in communication (e.g., not being able to find the ‘right’ words, camouflaging, poor communication between clients and clinicians, dismissal, exclusion from autistic spaces)
7. Systemic barriers (e.g., delays, service fragmentation, financial barriers, absent or overburdened services)

Grade-CerQUAL Summary of Findings and Evidence Profile

Table S.8

*Grade-CerQUAL Summary of Findings and Evidence Profile*

| **Findings** | | **Studies contributing to findings** | **Methodological concerns (based on JBI quality assessment)** | **Coherence** | **Relevance** | **Adequacy** | **Assessment of confidence in the evidence** |
| --- | --- | --- | --- | --- | --- | --- | --- |
| Subtheme 1: Recognition and decision-making | 1.1 Autistic people and their family members often struggled to identify their needs and then decide to seek support. | Atherton et al. (2022), Cage et al. (2024), de Broize et al. (2021), Evans (2022), Freeman & Paradis (2023), Ghanouni & Seaker (2023), Leedham et al. (2020), Lewis (2017) | Minor concerns. Some contributing studies did not report on the researcher’s influence or positionality. | No or very minor concerns. Included studies detailed various challenges in noticing one’s needs and then deciding to seek support. | Minor concerns.  Clinician perspectives were not represented in this finding, but this finding directly relates to the review question. | No or very minor concerns. There was a large amount of relevant data from quotes and author interpretations. | High confidence |
|  | 1.2 Many autistic people had previously sought support for their mental health and received diagnoses they felt did not adequately explain their experiences. This caused mistrust and discouraged help-seeking. | Ardeleanu et al. (2024), Cumin et al. (2021), Darling Rasmussen (2023), Leedham et al. (2020), Lewis (2017), Pearse (2020), Punshon et al. (2009), Raymond-Barker et al. (2016), Smith (2021), Tamilson et al. (2024), Wigham et al. (2022). | Minor concerns. Some contributing studies did not report on the researcher’s influence or positionality. Case series did not always justify case selection. | No or very minor concerns. Included studies clearly reflected the effects of past misdiagnosis on seeking support. However, there were some participants who had never sought help from the mental health care system before.  We explore the effects of co-occurring conditions in 3.1. | No or very minor concerns. Includes studies from all three perspectives. A range of conditions is named as a misdiagnosis. This finding directly relates to the review question. | No or very minor concerns. There was a very large amount of relevant data from quotes and author interpretations. | High confidence |
|  | 1.3 The decision to seek a diagnosis often depended on an appraisal of its perceived benefits (e.g., access to services) and costs (financial and emotional demands, potential for discrimination) | Ardeleanu et al. (2024), Cage et al. (2023), Cuda Pierce (2022), de Broize et al. (2022), Lam et al. (2024), Lewis (2017), Raymond-Barker et al. (2016), Tien et al. (2025). | Minor concerns. Some contributing studies did not report on the researcher’s influence or positionality. Case series did not always justify case selection. | Minor concerns. Participants expressed different views regarding the value of a diagnosis and the perception of available supports and services. We describe these instances of disagreement. | Minor concerns.  Clinician perspectives were less represented in this finding, but this finding directly relates to the review question. | No or very minor concerns. There was a large amount of relevant data from quotes and author interpretations. | Moderate confidence |
|  | 1.4 Autistic people and family members often relied on insights from those with lived experience when seeking a diagnosis or post-diagnostic supports. This extended to which services were selected and how they were approached. | Atherton et al. (2022), Cumin et al. (2021), de Broize et al. (2022), Gerhand (2020), Huang et al. (2022), Lewis (2017), Murphy et al. (2022), Raymond-Barker et al. (2016), Seers & Hogg (2022), Tien et al. (2025). | Minor concerns. Some contributing studies did not report on the researcher’s influence or positionality. | Minor concerns. Included studies explored how autistic people sought out information from the autistic community. However, there was some discordance regarding post-diagnostic support groups, which we explore. | Moderate concerns. Includes data from the three stakeholder groups. This finding is likely less relevant to countries where public healthcare assessments are not available or where access to local autistic-led resources is limited. This finding directly relates to the review question | No or very minor concerns. There was a large amount of relevant data from quotes and author interpretations. | Moderate confidence |
| Subtheme 2: Negotiation at the interface | 2.1 Past negative experiences with stigmatisation and/or misdiagnosis when seeking help discouraged many autistic people’s from seeking a diagnosis. | Ardeleanu et al., (2024), Atherton et al., (2022), Evans, (2022), Legg et al. (2023), Lewis (2017), Murphy et al. (2023), Punshon et al. (2009), Tien et al. (2025). | Minor concerns. Some contributing studies did not report on the researcher’s influence or positionality. | No or very minor concerns. Included studies explored this finding in detail, as seen in quotes and author interpretations. A large quantitative survey (Lewis, 2017) supported the finding, | Minor concerns. Clinicians alluded to mistrust in 2.7 but this finding was mostly relevant to autistic people and their family members. This finding directly relates to the review question. | No or very minor concerns. There was a large amount of relevant data from quotes and author interpretations. | High confidence |
|  | 2.2 Obtaining a referral was often challenging due to gatekeeping or lack of awareness. | Ardeleanu et al. (2024), Atherton et al. (2022), Evans (2022), Friedman et al. (2024), Hayes et al. (2021), Lewis (2017), Murphy et al. (2023), Pearse (2020), Raymond-Barker et al. (2016), Seers & Hogg (2022), Stagg & Belcher (2019), Tamilson et al. (2024), Wigham et al. (2022). | Minor concerns. Some contributing studies did not report on the researcher’s influence or positionality. | No or very minor concerns. Included studies explored this finding in detail, as seen in quotes and author interpretations. | No or very minor concerns. All three stakeholder groups explored the challenges faced in obtaining referrals. This finding directly relates to the review question. | No or very minor concerns. There was a very large amount of relevant data from quotes and author interpretations. | High confidence |
|  | 2.3 The diagnostic process sometimes came with an emotional weight and a fear of not receiving a diagnosis, which served as deterrents. | Atherton et al. (2022), Cage et al. (2023), Cuda Pierce (2022), Cumin et al. (2021), Pearse (2020). | Minor concerns. Some contributing studies did not report on the researcher’s influence or positionality. | No or very minor concerns. Included studies explored this finding in depth, as seen in quotes and author interpretations. | Minor concerns. Clinicians and autistic people recognised the emotional weight of the expectations, but there was no relevant data from family members. This finding may be less relevant to those with a less strong sense of autistic identity. This finding directly relates to the review question. | Minor concerns. This finding was informed by relatively less data compared to others, although the data was judged to be sufficient to warrant being reported separately. | Moderate confidence |
|  | 2.4 Many autistic people’s communication was misinterpreted, whether because they struggled to express their concerns in ways that clinicians would interpret correctly or because their communication appeared more typical. | Cuda Pierce (2022), Darling Rasmussen (2023), Fiori Nastro et al. (2023), Lewis (2017), Murphy et al. (2023), Punshon et al. (2009), Smith (2021) | Minor concerns. Some contributing studies did not report on the researcher’s influence or positionality. Case series did not always justify case selection. | No or very minor concerns. Included studies explored this finding in depth, as seen in quotes and author interpretations. Experiences of misinterpretation were reported consistently. | Moderate concerns. This finding was expressed by autistic people and clinicians but not family members. This finding directly relates to the review question. | No or very minor concerns. There was a very large amount of relevant data from quotes and author interpretations, as well as quantitative data. | High confidence |
|  | 2.5 Camouflaging sometimes hindered the referral and diagnostic process and made it harder to access post-diagnostic supports. | Cage et al. (2023), Craddock (2023). Cumin et al. (2021), Dodd (2021), Gonçalves-Garcia et al. (2024), Lam et al. (2024), McQuaid et al. (2022). Murphy et al. (2022). | Minor concerns. Some contributing studies did not report on the researcher’s influence or positionality. Case series did not always justify case selection. | Moderate concerns. Although included studies provided appropriate quotes and author interpretations, the quantitative data on the association between camouflaging and late diagnosis was inconclusive and its contribution was unclear. | Moderate concerns. This finding was expressed by autistic people and clinicians but not family members. This finding directly relates to the review question. | Moderate concerns. With regards to camouflaging during the assessment, the qualitative data was relatively less rich compared to camouflaging prior to the assessment. | Moderate confidence |
|  | 2.6 Autistic people and clinicians recognised the limitations of the diagnostic process, especially in relation to adult women. Some rejected the idea of a clinical diagnosis altogether. | Ardeleanu et al. (2024), Cage et al. (2024), Craddock (2024), Cumin et al. (2021, Darling Rasmussen (2023), de Broize et al. (2022), Friedman et al. (2024), Hayes et al. (2021), Murphy et al. (2022), Seers & Hogg (2022). | Minor concerns. Some contributing studies did not report on the researcher’s influence or positionality. | No or very minor concerns. Included studies explored this finding in depth, as seen in quotes and author interpretations. | Minor concerns. Clinicians and autistic people agreed about the limitations of diagnostic tools and criteria, although family members did not provide any relevant data. Rejecting the ‘medicalisation’ of autism was a less represented perspective. | No or very minor concerns. There was a large amount of relevant data from quotes and author interpretations. | High confidence |
| 3. Resistance and Resolutions | 3.1 The presence of co-occurring conditions complicated autism diagnosis due to diagnostic overshadowing or the overlap between autistic traits and symptoms of other conditions. The presence of an informant was beneficial in many cases, | Cumin et al. (2021), Darling Rasmussen (2023), de Broize et al. (2022), Fiori Nastro et al. (2022), Freeman & Paradis (2021), Gilmore & Hayes (1999), Gonçalves-Garcia et al. (2024), Hayes et al. (2021), Heijnen-Kohl et al. (2022), Jadav & Bal (2021), McKenzie et al. (2015), Punshon et al. (2009), Roy & Balaratnasingam (2010), Smith (2021), van Niekerk et al. (2011). | Minor concerns. Some contributing studies did not report on the researcher’s influence or positionality. Case series did not always justify case selection. | No or very minor concerns. Included studies explored this finding in detail, as seen in quotes and author interpretations. | Minor concerns. All three stakeholder groups reported on this finding. A wide variety of conditions and settings was reported on. The data on the role of informants was inconsistent and the surrounding difficulties were not explored in detail. | No or very minor concerns. There was a large amount of relevant data from quotes and author interpretations, as well as quantitative data. | High confidence |
|  | 3.2 Mental health crises could both trigger and delay an autism diagnosis. | Cumin et al. (2021), Darling Rasmussen (2023), Gerhand (2020), Heijnen-Kohl et al. (2022), Leedham et al. (2020), Tamilson et al. (2024), Tien et al. (2025). | Minor concerns. Some contributing studies did not report on the researcher’s influence or positionality. Case series did not always justify case selection. | Minor concerns. There were contrasting experiences with regards to the impact of mental health crises which are explored in the synthesis. | Moderate concerns. This finding only applied to few participants and family perspectives were not represented. | Moderate concerns. This finding was informed by fewer studies and less data compared to others, although the data was judged to be sufficient to warrant being reported as a separate finding. | Moderate confidence |
|  | 3.3 It was very challenging to find appropriate providers and diagnostic and post-diagnostic services. Accessible, reliable, and appropriate information about diagnostic and post-diagnostic services was scarce or difficult to locate. | Ardeleanu et al. (2024), Cage (2023), de Broize et al. (2022), Evans (2022), Lam et al. (2024), Legg et al. (2023), Lewis, (2017), Scattoni et al. (2021), Wigham et al. (2022, 2023) | Minor concerns. Some contributing studies did not report on the researcher’s influence or positionality. Case series did not always justify case selection. | Minor concerns. Autistic people and family members clearly articulated their experiences with inadequate service provision across contributing studies, though clinician voices were less represented, There was some disagreement between clinicians and autistic people/family members in relation to information accessibility. | No or very minor concerns. All three stakeholder groups reported on this finding. A wide variety of settings and services was reported on. | No or very minor concerns. There was a large amount of relevant data from quotes and author interpretations, as well as quantitative data. | Moderate confidence |
|  | 3.4 Autistic people and their families faced long waitlists. Services, where available, were often fragmented, inadequately coordinated, or inappropriately siloed. | Ardeleanu et al. (2024), Cage et al. (2023), Darling Rasmussen (2023), Evans (2022), Lam et al. (2024), Legg et al. (2023), Lewis (2017), Raymond-Barker et al. (2016), Wigham et al. (2022). | Minor concerns. Some contributing studies did not report on the researcher’s influence or positionality. Case series did not always justify case selection. | No concerns. All three stakeholder groups clearly articulated their experiences with undue waitlists and service siloing across contributing studies. | No or very minor concerns. All three stakeholder groups reported on this finding. A wide variety of settings and services was reported on. | No or very minor concerns. There was a large amount of relevant data from quotes and author interpretations, as well as quantitative data. | High confidence |
|  | 3.5 Systemic and logistical barriers, such as financial and geographical factors, hindered access to diagnosis and post-diagnostic services. | de Broize et al., (2022); Ghanouni & Seaker (2023), Hamdani et al. (2023), Huang et al. (2022), Murphy et al., (2022), Raymond-Barker et al. (2016), Wigham et al. (2022), Wigham et al., (2023). | Minor concerns. Some contributing studies did not report on the researcher’s influence or positionality. | No concerns. All three stakeholder groups clearly articulated their experiences across contributing studies. | No or very minor concerns. All three stakeholder groups reported on this finding. A wide variety of settings was reported on. | No or very minor concerns. There was a large amount of relevant data from quotes and author interpretations, as well as quantitative data. | High confidence |
|  | 3.6 Even after a diagnosis, many autistic people and their families struggled to get the right support, because their needs were perceived as invisible. | Craddock (2023), Finch et al. (2022), Huang et al. (2022b), Leedham et al. (2020), Raymond-Barker et al. (2018). | Minor concerns. Some contributing studies did not report on the researcher’s influence or positionality. | No concerns. All three stakeholder groups clearly articulated their perspective on service coordination across contributing studies, although clinicians seemed to have more ‘insider knowledge’. | No or very minor concerns. All three stakeholder groups reported on this finding. A wide variety of settings was reported on but limited to Western settings. | No or very minor concerns. There was a large amount of relevant data from quotes and author interpretations, as well as quantitative data. | High confidence |
